# Comparing loss of individual fragile X proteins suggests strong links to cellular senescence and aging

**DOI:** 10.1007/s00018-025-05898-0

**Published:** 2025-10-21

**Authors:** Sonja Menge, Inmaculada Segura, Max Hartmann, Lorena Decker, Selin Kiran, Karin M. Danzer, Sebastian Iben, Angelika B. Harbauer, Patrick Oeckl, Axel Freischmidt

**Affiliations:** 1 Department of Neurology, Ulm University, ZBMF, Helmholtzstr. 8/1, Ulm, 89081 Germany; 2 Max Planck Institute for Biological Intelligence, Martinsried, Germany; 3 Biomedical Center Munich, Department of Cellular Physiology, Ludwig-Maximillian- University, Martinsried, Germany; 4 Department of Dermatology and Allergic Diseases, Ulm University, Ulm, Germany; 5 German Center For Neurodegenerative Diseases (DZNE) Ulm, Ulm, Germany; 6 School of Medicine and Health, Institute of Neuronal Cell Biology, Technical University of Munich, Munich, Germany; 7 Munich Cluster for Systems Neurology, Munich, Germany

**Keywords:** Alzheimer’s disease, Parkinson’s disease, Amyotrophic lateral sclerosis, Fragile x syndrome, Protein aggregation

## Abstract

Members of the fragile X protein (FXP) family (FMR1, FXR1 and FXR2) are differentially expressed in most types of cancer and major neurodegenerative diseases. While increased expression of FXR1 in cancer has been linked to senescence evasion and consequently tumor initiation and progression, decreased expression of FXPs in neurodegeneration may contribute to pathogenic protein aggregation and death of vulnerable neurons. However, due the causal role in fragile x syndrome, most data are available about loss of FMR1 in neurons while functions of FXR1 and especially FXR2 remain largely unexplored. To address this knowledge gap, and to directly compare functions of the FXPs, we used proteomics of CRISPR/Cas9 edited HAP1 cells carrying knockouts of the individual FXPs for identification of cellular mechanisms associated with these proteins. Further exploration of proteomic findings suggests roles of the FXPs in ribosome biogenesis, autophagy and mitochondrial health linked to organismal aging, and cellular senescence. Validation of FXP induced defects relevant for neurodegenerative diseases in neuroblastoma cell line SH-SY5Y upon FXP knockdown revealed high cell type specificity of individual FXP functions. Overall, we provide a comprehensive overview and comparison of cellular mechanisms related to the individual FXPs, as well as starting points for further studying this protein family in respective cell types of FXP associated diseases, and in aging in general.

**Supplementary Information:**

The online version contains supplementary material available at 10.1007/s00018-025-05898-0.

## Introduction

The Fragile X protein (FXP) family comprising FMR1 (or FMRP) and its homologues FXR1 and FXR2 are multifunctional RNA-binding proteins involved in numerous diseases, such as neurodevelopmental, neurodegenerative and psychiatric disorders, but also myopathies, premature ovarian insufficiency, and cancer. These proteins are well-known for their role in suppressing protein synthesis during RNA transport, and thereby regulating local translation. However, the FXPs have been linked to multiple additional cellular processes, dependent on their RNA-binding properties and/or protein-protein interactions. These include most aspects of RNA metabolism, i.e. transcription, splicing, editing, nuclear export, transport, stability and translation of mRNAs, but also involvement in DNA damage and stress responses, mitochondrial organization, cell cycle regulation, ribosome biogenesis and gating of ion channels. It is noteworthy that most of our knowledge about this protein family is derived from studying FMR1, whereas relatively little is known about FXR1 and especially FXR2 [1–3].

The best-known disease genetically linked to the FXPs is the neurodevelopmental disorder Fragile X syndrome (FXS), the leading inherited cause of intellectual disability and autism spectrum disorders. Here, a trinucleotide (CGG) repeat expansion (> 200 repeats) in the promoter/5’ untranslated region (UTR) of *FMR1* leads to its hypermethylation and silencing of expression, resulting in dysregulation of predominantly synaptic transcripts and impaired synaptic plasticity [4]. Intermediate CGG repeat lengths in *FMR1* (55–200 repeats) are responsible for expression of aberrant *FMR1* mRNA and protein and are associated with Fragile X–associated premature ovarian insufficiency (FXPOI) in women and, primarily in men, with the neurodegenerative disease Fragile X–associated tremor/ataxia syndrome (FXTAS) [5]. Furthermore, *FXR1* and *FMR1* have been genetically and/or functionally linked to psychiatric diseases, i.e. schizophrenia, bipolar disorders and mood regulation [1, 6, 7], and recessive mutations in muscle-specific exon 15 of *FXR1* are causative for congenital myopathies with varying severity, depending on the underlying mutation [8]. Evidence for the contribution of FXR2 to human diseases is lacking so far, but phenotypes of *Fxr2* knockout mice partly overlap with *Fmr1* knockout mice, and suggest an important role of this protein in the central nervous system [9]. Besides diseases mentioned above, there is increasing evidence for contribution of all three FXPs to major neurodegenerative diseases and cancer.

Loss of expression of individual FXPs has been reported in different neurodegenerative diseases, namely Alzheimer’s disease (AD), Parkinson’s disease (PD) and amyotrophic lateral sclerosis (ALS). In AD, FMR1 regulates the translation of amyloid precursor protein (*APP)* mRNA, and loss of FMR1 leads to overexpression of APP contributing to the deposition of characteristic amyloid-beta (Aß) plaques [10, 11]. In PD, loss of FMR1 was evident before occurrence of Lewy body pathology in the substantia nigra pars compacta of PD patients, and in patients suffering from early-stage incidental Lewy body disease. Notably, overexpression of α-synuclein led to decreased FMR1 levels in both cultured dopaminergic neurons and mouse brains [12].

Most data about the FXPs in neurodegeneration are available from studying ALS. Here, the FXPs interact with several proteins encoded by established ALS genes, and RNA targets of the FXPs are enriched in ALS-related mRNAs and microRNAs [3, 13, 14]. Moreover, the single *Drosophila* homologue of the FXP family, dFMR1, was identified to modify the toxicity of TDP-43 [15]. In *post mortem* spinal cord tissue of ALS patients, aberrant expression of FXR1 and FXR2 were detected independently of the underlying disease gene, and analyses of rare ALS cases caused by mutations in *FUS* suggested occurrence of neuronal cytoplasmic FUS inclusions in motoneurons that lost expression of FXR1 and especially FXR2 [14]. Single-cell proteomics detected downregulation of FXR1 and FXR2 in motoneurons of sporadic ALS patients, suggesting a link of these proteins also to the almost universal TDP-43 pathology in ALS [16]. Strikingly, two different animal models indicate an important functional role of the FXPs in ALS pathogenesis. In a *Drosophila* TDP-43 model of ALS, overexpression or knockdown of dFMR1 substantially mitigated or worsened, respectively, the phenotype characterized by eye depigmentation, increased larval turning times, and severely reduced life span. Moreover, overexpression of dFMR1 improved the solubility of TDP-43 [15]. In a zebrafish model of FUS-linked ALS, overexpression of human FMR1 along with mutant FUS fully rescued FUS-induced locomotor defects [13]. In both models, molecular analyses were limited, but (d)FMR1 rescued neuromuscular junction pathology, likely by normalizing local expression of MAP1B (or its homologue *futsch* in *Drosophila*).

The role of the FXPs in cancer is the exact opposite to the loss of expression and/or function in neurodegenerative diseases. Increased expression of FMR1, FXR2 or especially FXR1 in most types of cancer [17, 18] has been linked to dysregulation of oncogenes and tumor suppressors, and contributes to key cancer hallmarks such as cell proliferation, metastasis, epithelial-mesenchymal transition, and angiogenesis [19–23]. High levels of FXR1 block cellular senescence [22, 24–27], a crucial tumor-suppressing mechanism, and consequently further facilitate tumor initiation and progression [1].

Despite the involvement of all three FXPs in various diseases, there is a strong bias in studying FMR1, while the functions of FXR1 and especially FXR2 remain largely unexplored. Furthermore, studies directly comparing functions of FMR1, FXR1 and FXR2 are very rare. To address this gap, we used unbiased proteomics of HAP1 cells carrying knockouts of the individual FXPs, and compare downstream events upon loss of FMR1, FXR1 or FXR2.

## Materials and methods

### Cell lines and cell culture

CRISPR/Cas9-edited HAP1 cell lines carrying short out-of-frame deletions in *FMR1*, *FXR1* or *FXR2* were purchased from Horizon Discovery (Waterbeach, UK), and are listed in Supplementary Table S1. SH-SY5Y cells were bought from the American Type Culture Collection (#CRL-2266). HAP1 and SH-SY5Y cells were grown under standard conditions in in IMDM and DMEM, respectively, supplemented with 10% fetal calf serum. Passage numbers of both HAP1 and SH-SY5Y cell Lines were between 5 and 19 throughout the study. The different HAP1 cell lines were always grown in parallel, and all results reported here are based on comparing cell lines of identical age.

### Transfection and plasmids

HAP1 cells were transfected using calcium phosphate precipitation as described [28], with minor modifications. Solely for the NanoLuciferase (NanoLuc) assay measuring translational fidelity of ribosomes, electroporation was used for transfection (see below). Transfection of plasmids coding for shRNAs in SH-SY5Y cells were performed with the Effectene Transfection Reagent Kit (Qiagen, #301425) according to the manufacturer’s protocol. Sequences of shRNAs used are listed in Supplementary Table S2.

All plasmids used in this study were verified by Sanger sequencing, and are listed in Supplementary Table S3.

### Preparation of total and insoluble proteins for proteomics

Untreated HAP1 cells were thoroughly washed in PBS 48 h after seeding. For total protein, cells were lysed in urea buffer (20 mM Tris, 8 M urea, pH 8), briefly sonicated and stored at −80° C. For insoluble protein, cells were harvested in PBS and lysed by sonication. Lysates were then centrifuged for 30 min at 100,000 x *g* and 4° C, followed by resuspension of protein pellets in PBS by sonication. After another centrifugation for 30 min at 100,000 x *g* and 4° C, washed protein pellets were resuspended in urea buffer and stored at −80° C.

### Label-free proteomic analysis of HAP1 total and insoluble protein

Total and insoluble protein preparations (see above) containing 100 µg protein were filled to 200 µl containing a final concentration of 100 mM triethylammonium bicarbonate (TEAB), 10 mM Tris(2-carboxyethyl)phosphine hydrochloride (TCEP) and 40 mM 2-chloroacetamide, and were incubated for 60 min at 60° C for reduction and alkylation. Samples were buffer exchanged with 50 mM TEAB and digested with trypsin/LysC (Promega, protein-to-enyzme ratio 50:1) using a filter-aided sample preparation protocol [29]. Peptides were fractionated using STAGE Tips (AttractSPE Disks Bio SDB, Affinisep) into 3 fractions using 15% acetonitrile (ACN, fraction 1), 24% ACN (fraction 2) and 70% ACN in 20 mM NH_4_-Formiat (pH 10). The fractions were vacuum dried and resuspended in 12 µl 0.5% trifluoroacetic acid (TFA). Peptides were separated using an UltiMate 3000 RSLCnano system and a PepMap100 C18, 20 × 0.075 mm, 3 μm trap column (Thermo Fisher Scientific) and a PepMap100 C18, 50 × 0.050 mm, 2 μm analytical column (Thermo Fisher Scientific). Mobile phase of the loading pump (trap column) was 0.05% TFA/2% Methanol (flow rate: 5 µl/min), the mobile phase of the nano pump (analytical column) was A: 4% DMSO/0.1% formic acid, and B: 4% DMSO/76% acetonitrile/0.1% formic acid (flow rate: 150 nl/min) and peptides were separated with a 3 h gradient. Peptides were infused into a QExactive mass spectrometer (Thermo Fisher Scientific) and measured with data-dependent acquisition (Top15). Proteins were identified using MaxQuant 1.6.17.0 and the human reference proteome from UniProt (downloaded December 1 st, 2020) using default settings with carbamidomethylation as fixed modification and methionine oxidation and N-terminal acetylation as variable modifications. An FDR of 1% was used for peptide and protein identification and protein quantification was performed with the MaxLFQ algorithm [30].

### Proteomic data analyses

To ensure optimal comparability, only proteins with LFQ intensities available in at least 50% of samples (two out of four) from each cell line were considered for all downstream analyses. Proteins showing minimal variation (< 30%) compared to the control cell line were excluded, and a one-way ANOVA was used to identify differentially expressed proteins. After adjusting ANOVA *p*-values for multiple testing [31], *post hoc* Tukey HSD was used to detect significant differences between the groups and to correct *p*-values for multiple comparisons. Enrichment analyses of differentially expressed proteins was performed using the Enrichr database [32, 33]. For principal component analyses (PCA), we used ClustVis [34] with standard settings.

### Immunocytochemistry and Lipofuscin autofluorescence

Immunocytochemistry (ICC) was performed as described elsewhere [14]. Autofluorescent lipofuscin accumulations were detected using high laser intensities (excitation 488 nm, emission 525 nm) as reported previously [35].

### Live cell imaging

For dyes requiring live cell imaging, cells were seeded in glass bottom dishes (µ-Dish 35 mm, high Glass Bottom, IBIDI, #81158) coated with poly-D-lysine (PDL). Cells were washed with PBS once before application of specific dyes (Lysotracker™ Green DND-26, Thermo Fisher Scientific, #L7526; JC-1 Mitochondrial Membrane Potential Assay Kit, abcam, #ab113850; DCFDA/H2DCFDA - Cellular ROS Assay Kit, abcam, #ab113851) according to the recommendations of the manufacturer. Hoechst Nuclear Stain 33342 (5 µg/ml final concentration) was added 15 min before the end of the incubation time of the respective dye, followed by washing the cells with PBS. For imaging, cells were covered with 200 µl 10% fetal calf serum in PBS to maintain cell survival during imaging.

For Live cell imaging of mitophagy and videos for mitochondrial motility, cells were cultured in 24-well glass Bottom Black Plates (Cellvis, #P24-1.5 H-N). For imaging, growth medium was replaced by phenol-red free media (Hibernate E minus phenol red, Transnet by Brain Bits, #HEPR500).

### Microscopy

Confocal microscopy (ICC, lipofuscin and Lysotracker/JC-1/ROS live cell imaging) was performed with the laser-scanning microscope ZEISS LSM 980 with Airyscan 2 (Axio Observer.Z1/7) with a Plan-Apochromat 100x/1.40 Oil M27 objective using the Zen blue software (version 3.3).

For confocal microscopy of mitophagy and for recording videos of mitochondrial motility, the Eclipse Ti2 spinning-disk microscope equipped with a DS-Qi2 high-sensitivity monochrome camera using a ×60 NA 1.40 oil-immersion lens and NIS-Elements software (Nikon) was used. Videos to track mitochondrial motility were recorded with the following time schedule: 1 s interval, 40 s duration, 41 loops.

All confocal microscopy settings, including laser intensities, photomultiplier tube (PMT) voltage, pinhole size, and gain, were kept constant within each individual experiment.

### Image and video analyses

All image analyses were carried out on original, unaltered images to preserve data integrity using Fiji (2.14.0 release, 32 bit, 2017 May 30) [36]. For quantification of fluorescence intensities, consistent thresholds across all images from the same experimental dataset for automated selection of regions-of-interest were used to minimize bias. To account for fluctuations in fluorescence intensities, data were normalized to control cells in each independent experiment. Granule/vesicle/puncta counting was performed using the ‘*Analyze Particles*’ or ‘*Find Maxima’* functions in Fiji. Here too, consistent thresholds for automated counting were used across all images from the same independent experiment to minimize bias. Colocalization analyses were performed using the Fiji JACoP v2.0 plugin 2009 [37]. The Manders’ Coefficients were used to estimate overlap between respective channels within regions-of-interest. Image analyses was either performed at the level of single cells, or at the level of non-overlapping images from the same slide, whereby the mean of quantifiable cells (i.e. only complete cells) per image was used for further calculations. The figure legends indicate if data points refer to single cells, or to the mean of ≈ 5–20 cells from the same image.

For video-quantification of mitochondrial motility, we used the Fiji plugin “QuoVadoPro“ as described [38].

### Antibodies

All primary and secondary antibodies used for Western blotting and ICC are listed in Supplementary Table S4, including manufacturer, catalogue number and working concentration used.

### Additional kits and dyes

Kits and dyes that were used in this study according to the instructions of the manufacturer are listed in Supplementary Table S5. Potential modifications of the standard protocol are indicated.

### Nanoluciferase assay for translational fidelity of ribosomes

The NanoLuc assay for measuring the translational fidelity of ribosomes was performed exactly as previously described [39].

### Fractionation of soluble and insoluble proteins for Western blotting

HAP1 cells were thoroughly washed with PBS and lysed in RIPA buffer (50 mM Tris, 150 mM sodium chloride, 0.25% sodium deoxycholate, 1% NP-40, 1 mM EDTA, pH 7.4). Lysates were briefly sonicated and adjusted to a protein concentration of 1 mg/ml. Next, 200 µl of lysates were centrifuged at 100,000x *g* and 4° C for 30 min, and resulting supernatants represent the soluble fraction. Pellets of insoluble protein were washed with RIPA buffer and centrifuged again at 100.000x *g* and 4° C for 30 min. Protein pellets were then resuspended in 200 µl 8 M urea buffer (20 mM Tris, 8 M urea, pH 8.0), and represent the insoluble fraction. Equal volumes (20 µl) of soluble and insoluble proteins were used for Western blotting.

### Flow cytometry

Flow cytometry was performed as described [40]. Briefly, 250,000 cells per condition were resuspended in 500 µl Hibernate E minus phenol red (Transnet by Brain Bits, #HEPR500) and stained with 0.05 µM TMRE for 10 min. As a control for mitochondrial membrane disruption, control cells were treated with 10 µM CCCP for 10 min alongside TMRE. Fluorescent intensities were measured with a flow cytometer (Thermo Fisher Attune NxT), with the following settings: FSC 70 V, SSC 340 V, and YLA-1 280 V. Per condition 10,000 events were recorded, with a sample volume of 100 µl and a flow rate of 200 µl/min. Gates were set for live YL1-positive singlets (TMRE-stained cells). Data were normalized to the median fluorescence intensity (MFI) of YL1 singlets from untreated and TMRE-stained controls.

### RNA isolation and quantitative real-time PCR (qRT-PCR)

Cells were washed with PBS and harvested in RNAzol RT (Sigma-Aldrich, #R4533), and total RNA was extracted using the Direct-zol RNA Miniprep kit (Zymo Research, #R2050) according to the manufacturer’s protocol. Reverse transcription was performed with the QuantiTect Reverse Transcription Kit (Qiagen, #205311). qRT-PCRs were run in duplicates on a CFX96 real-time system (Bio-Rad) using the QuantiTect SYBR Green PCR Kit (Qiagen, #204143) and QuantiTect Primer Assays (Supplementary Table S6). For 47 S rRNA, oligonucleotides For: *TGTCAGGCGTTCTCGTCTC* and Rev: *GAGAGCACGACGTCACCAC* [41] were used in a final concentration of 200 nM each. C_t_-values were converted to Linear comparisons relative to the controls and normalized to endogenous TBP using the 2^−ΔΔCt^-method [42].

### Statistical analysis

Statistical analyses of proteomic data are described above. For all other experiments, a one-way ANOVA followed by *post hoc* Šídák’s test, or an unpaired, two-tailed Student’s *t*-test was used to detect significant differences between groups > 2 or = 2, respectively. *P*-values < 0.05 were considered statistically significant. Statistical analyses and generation of graphs was performed with Graphpad Prism software (version 8.4.3).

## Results

### The FXPs are involved in diverse cellular processes

FXP expression is altered in various types of cancer originating from multiple tissues and cell types [17, 18], as well as in neurologic [3, 4] and rare muscular diseases [8]. While the loss of FMR1 is relatively well studied in neurons due to its causal role in FXS, much less is known about FXR1 and FXR2, or functions of all three proteins in non-neuronal cells. To comprehensively analyse FXP functions, and to compare downstream events associated with the loss of each individual FXP, we exemplary used non-neuronal CRISPR/Cas9 edited HAP1 cells carrying knockouts (ko’s) of FMR1, FXR1, or FXR2. HAP1 is an adherent, near-haploid, fibroblast-like cell line derived from chronic myelogenous leukemia cell line KBM-7 that no more expresses hematopoietic markers [43]. We chose this cell line as a model for cancer that may also be indicative for neurodegenerative processes, because some pathologies of neurodegenerative diseases are reflected in fibroblasts (e.g [44–46]). Additionally, we established shRNA-mediated knockdowns (kd’s; ≈50%) of these proteins in undifferentiated SH-SY5Y cells for validation of findings relevant for neurologic diseases in a cell line of neuronal origin (Supplementary Fig. S1). Western blotting indicated comparable expression of FXR1 in HAP1 and SH-SY5Y cells, while FMR1 and FXR2 were roughly 50% and 25%, respectively, lower expressed in SH-SY5Y cells (Supplementary Fig. S2).

Due to the involvement of the FXPs in translational regulation [1, 3], we chose label-free proteomics of HAP1 cells for unbiased identification of differentially expressed proteins (DEPs; Supplementary Table S7). Separate analysis of FXP expression revealed that loss of FXR1 led to increased expression of FMR1 (≈ 25%), and a trend (*p* = 0.09) towards higher expression of FXR2 (≈ 25%). The loss of FXR2 induced an increase of FMR1 (≈ 40%) and a slight decrease of FXR1 (≈ 10%), while loss of FMR1 did not change expression of both FXR1 and FXR2 (Supplementary Fig. S3).

**Fig. 1:**
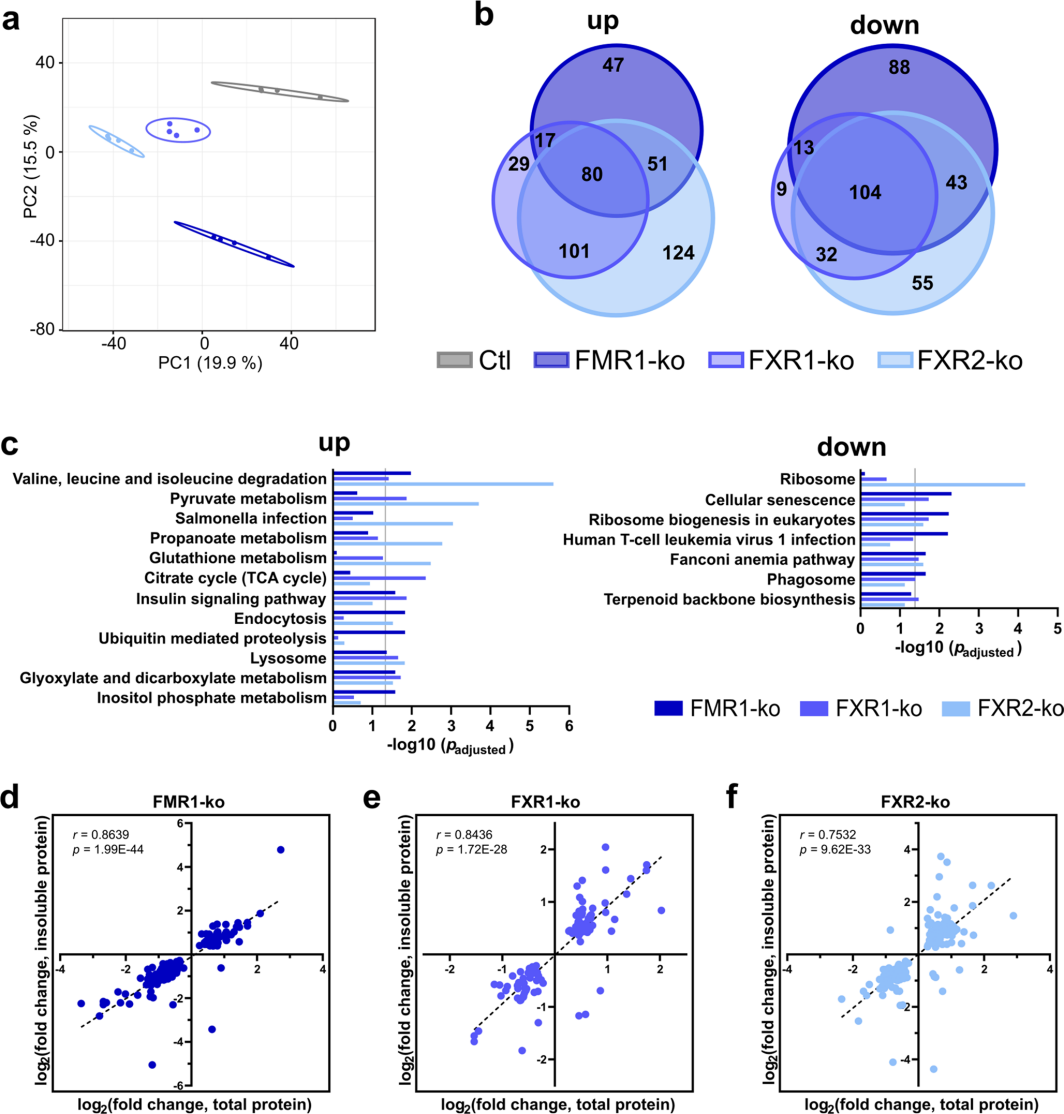
Loss of individual FXP family members leads to shared and unique proteomic changes. **a** PCA of 4656 proteins reliably quantified in proteomic analyses of HAP1 control (Ctl) and FXP-knockout (FXP-ko) cells. **b** Overlap of robustly up- and downregulated proteins. **c** KEGG pathway enrichment analyses of proteins with increased and decreased abundance, respectively. Top 5 KEGG pathways for each FXP including results for the other FXPs in the respective pathway are shown. Vertical lines indicate the significance threshold (*p*_*adjusted*_ = 0.05). **d-f** Correlation of differentially expressed proteins (DEPs) in the total and insoluble proteome of FXP-ko HAP1 cells (*n* = 4 for each cell line)

For optimal comparability of proteomic data, we restricted all analyses to 4656 proteins that were reliably detected in at least 50% of samples from each cell line. PCA clearly separated the different cell lines from each other (Fig. 1a) indicating unique changes associated with the loss of each individual FXP. Stringent criteria (fold change ≥ 30% compared to the control in any of the ko cells; ANOVA *p*_*adjusted*_ < 0.05; *post hoc* Tukey HSD) identified 523, 385 and 590 robustly DEPs in the FMR1-, FXR1- and FXR2-ko cells, respectively (Supplementary Table S7). Up- and downregulated proteins largely overlapped (Fig. 1b), but roughly 25%, 10% and 30% of dysregulated proteins were unique for the ko of FMR1, FXR1 and FXR2, respectively. KEGG pathway annotation of upregulated proteins revealed overrepresentation of proteins linked to different metabolic pathways, endocytosis and proteolysis. Downregulated proteins were enriched in proteins related to ribosomes and ribosome biogenesis, DNA damage repair, and cellular senescence (Fig. 1c; Supplementary Table S7). Overall, despite hundreds of aberrantly expressed proteins, enrichment of dysregulated proteins in specific pathways was rather weak.

In neurodegenerative diseases, the FXPs were repeatedly implicated in pathogenic protein aggregation (reviewed in [3]). Therefore, besides the total proteome, we performed analogue analyses of the insoluble proteome of these cells derived by centrifugation of cell lysates at 100,000 x *g*, and restricted all downstream analysis to 3802 proteins reliably quantified in all four cell lines. Here too, PCA clearly separated the cell lines from each other (Supplementary Fig. S4a), and 385, 330 and 655 robustly DEPs in the insoluble fraction of FMR1-, FXR1- and FXR2-ko cells, respectively, were identified (Supplementary Table S8; Supplementary Fig. S4b). Despite detected in the insoluble fraction, some proteins aggregating in neurodegenerative diseases, such as TDP-43 [47], were not increased in protein pellets of FXP-ko cells. However, KEGG pathway annotation of increased proteins revealed enrichment in proteasomal components as well as autophagic and lysosomal vesicles that may indicate defects in protein degradation pathways and/or protein folding. Additionally, KEGG terms of some neurodegenerative diseases were enriched, but these were largely driven by components of the proteasome. Decreased proteins showed very weak, if any, enrichment in specific pathways. An exception here were less abundant ribosomal proteins in FXR2-ko cells that were already detected in the total proteome (Supplementary Fig. S4c; Supplementary Table S8). Correlation of DEPs in the total and insoluble fraction of these cells indicates that the insoluble fraction is mostly a reflection of the total proteome (Fig. 1d-f), at least based on DEPs detected in both fractions.

### Loss of FXR1 or FXR2 impairs ribosome biogenesis in HAP1 cells

**Fig. 2:**
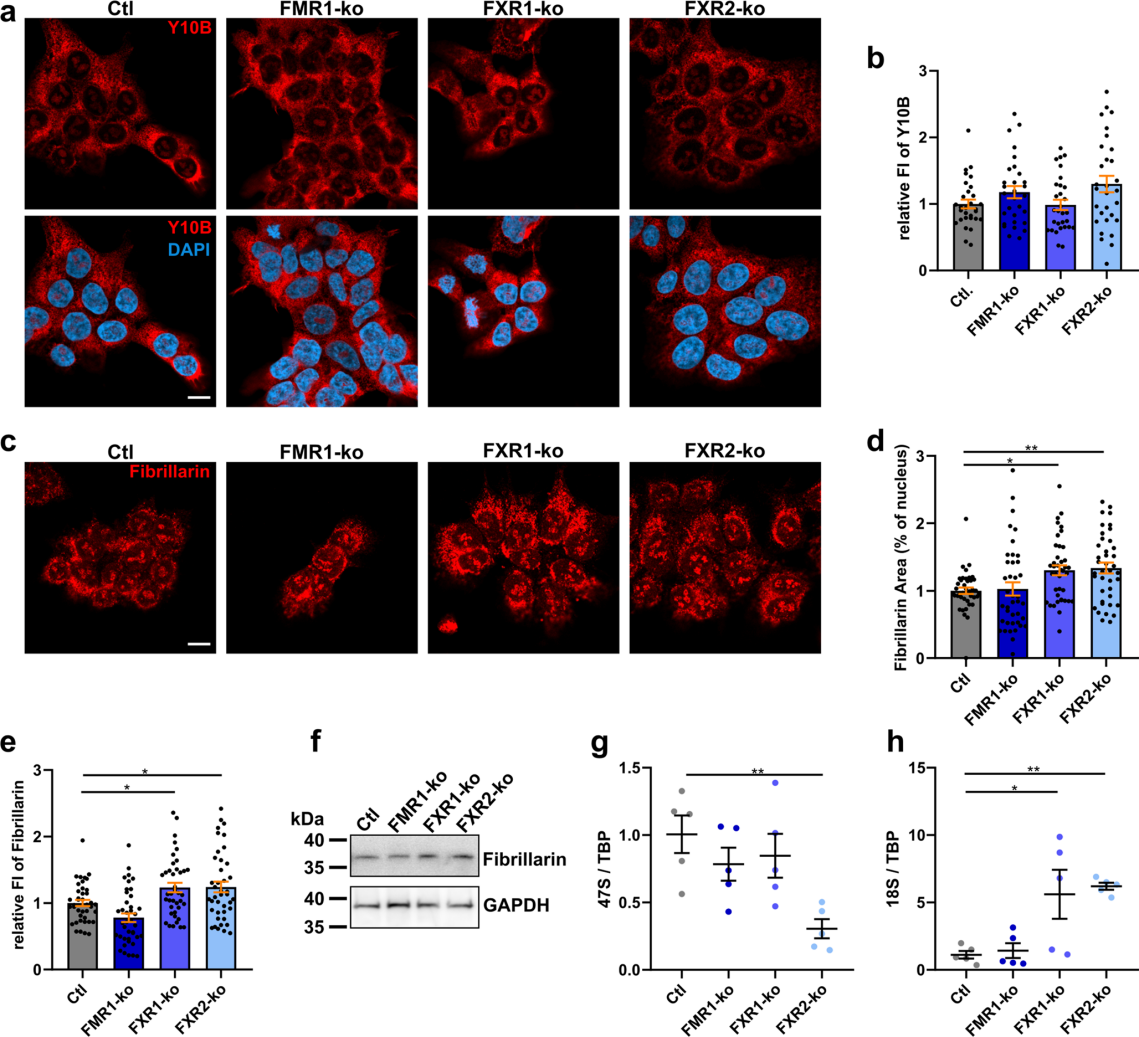
Loss of FXR1 or FXR2 leads to enlarged nucleoli and accumulation of 18 S rRNA in HAP1 cells. **a**,** b** Immunocytochemistry of mature 5.8 S rRNA (**a**; Y10B) and respective quantification of fluorescence intensities (**b**; *n* = 30 images from 3 independent experiments). **c-e** Representative images of Fibrillarin staining in HAP1 cells (**c**) and quantification of nucleolar areas (**d**) and Fibrillarin total fluorescence intensities (**e**; *n* = 39 images from 4 independent experiments). **f** Western blot of total Fibrillarin in HAP1 cells. **g**,** h** Relative abundance of 47 S pre-rRNA (**g**) and 18 S rRNA (**h**) measured by qRT-PCR. Data were normalized to TBP expression (*n* = 6; bars indicate mean ± SEM; *p < 0.05, **p < 0.01 in a one-way ANOVA followed by *post hoc* Šídák’s test; scale bars are 10 μm; FI = fluorescence intensity; TBP = TATA-box binding protein)

Proteomics indicated impaired ribosome biogenesis in all FXP-ko HAP1 cells (Fig. 1c). Interestingly, overproduction of ribosomes in hippocampal neurons of FMR1-ko mice was recently reported [48], and we used comparable methods to validate this finding in HAP1 cells. By staining Fibrillarin we determined nucleolar size that may reflect ribosome production, and Y10B antibody binding 5.8 S rRNA in mature 80 S ribosomes [49] to quantify total mature ribosomes. Additionally, we measured abundance of primary 47 S rRNA and 18 S rRNA to assess input and output, respectively, of ribosome biogenesis. Changes in the level of mature/precursors of rRNAs, including 18 S rRNA, may be indicative for defects in rRNA maturation and ribosome biogenesis. Abundance of primary 47 S rRNA may also be linked to defective ribosome biogenesis, and may additionally be indicative for more upstream defects, such as altered transcriptional activity and/or rDNA damage [50, 51]. While we could not detect any difference in the abundance of mature ribosomes (Fig. 2a, b), we found enlarged nucleoli upon ko of FXR1 or FXR2, but not FMR1 (Fig. 2c, d). Additionally, expression of total Fibrillarin was increased in FXR1- and FXR2-ko cells (Fig. 2e, f). Abundance of primary 47 S rRNA did not correlate with nucleolar size, and revealed decreased expression and/or increased turnover in FXR2-ko cells only (Fig. 2g). However, 18 S rRNA accumulated in FXR1- and, more robustly, in FXR2-, but not FMR1-ko cells (Fig. 2h). Hence, in HAP1 cells, loss of FXR1 or FXR2 lead to changes in ribosome biogenesis while loss of FMR1 had no effect, at least not on the markers measured here.

### Loss of FXPs does not lead to increased protein aggregation

Analyses of total and insoluble proteomes did not indicate increased protein misfolding/aggregation upon FXP loss in HAP1 cells (Fig. 1d-f). However, enrichment of components of protein degradation machineries in insoluble proteomes (Supplementary Fig. S4c) may be indicative for increased protein misfolding and/or aggregation. Addressing this issue, we used Proteostat Aggresome detection reagent to label larger accumulations of misfolded proteins. Here, FMR1-ko cells showed increased staining intensities (Supplementary Fig. S5a, b), but from proteomic data we could not define which proteins are accumulating (Fig. 1d). Therefore, to better characterize protein accumulation, we hypothesized that defects in proteostasis may cause protein accumulation, and took advantage of the established Fluc-EGFP system. Here, a conformationally instable firefly luciferase (Fluc) fused to EGFP is expressed, and formation of intracellular aggregates is indicative for proteostasis defects and availability of chaperones. By introduction of single (R188Q; FlucSM) and double (R188Q and R261Q; FlucDM) mutations, Fluc is further destabilized, resulting in proteostasis sensors of different sensitivities [52, 53]. However, when expressed in the FXP-ko cells, we could not detect increased aggregation of any of these sensors (Supplementary Fig. S5c-g).

Next, because of the well-defined role of the FXPs in regulating translation, we hypothesized that decreased fidelity of translation may lead to random amino acid exchanges resulting in misfolding of random proteins that are not detectable by proteomics. To test this hypothesis, we used a recently published assay where inactive variants of NanoLuc with either a stop-codon in the coding sequence (Y18X) or an amino acid exchange (R162S) are expressed in cells. Readthrough of stop-codons or amino acid exchanges reconstitute luciferase activity, and are indicative for translational infidelity [39]. Additionally, we used a dye fluorescing only when reacting with free cysteine exposed by misfolded proteins (TPE-MI; [54]). However, translation was more error-prone in FXR2-ko cells only (Supplementary Fig. S5h, i), and TPE-MI fluorescence intensities indicated even slightly less misfolded proteins in FMR1- and FXR1-ko cells (Supplementary Fig. S5j, k). Therefore, protein folding/aggregation is most likely not affected by FXP loss, and increased Proteostat intensities upon ko of FMR1 may reflect increased protein synthesis. For testing, we used established puromycin labelling [55] of proteins currently synthesized, and found increased protein synthesis in FMR1-ko cells only (Supplementary Fig. S5l, m). Thus, increased Proteostat intensities in FMR1-ko cells are most likely unrelated to protein misfolding and/or accumulation/aggregation, but may reflect excess protein synthesis and/or higher protein load.

Finally, to investigate if challenging FXP-ko cells with stress may reveal links to protein aggregation, we treated the cells with sodium arsenite (50 µM for 30 min), and assessed the solubility of TDP-43 by fractionating soluble and insoluble proteins followed by Western blotting. While we detected the well-known decrease in TDP-43 solubility upon application of stress, this was comparable between the cell lines (Supplementary Fig. S5n, o). Taken together, from our data we conclude that loss of individual FXPs is not related to increased protein aggregation under basal conditions, and has no effect on the solubility of TDP-43 even after application of stress.

### FXP loss impairs autophagy but not proteasomal protein degradation

Concerning possibly impaired protein degradation, we measured proteasomal activity in lysates of FXP-ko cells using a fluorescent substrate, and determined global protein ubiquitination by Western blotting. Both assays did not reveal any differences between the cell lines (Supplementary Fig. S6). Consequently, we also addressed ubiquitin-independent proteasomal protein degradation by expressing EGFP or EGFP fused to the 44 C-terminal amino acids of mouse Ornithine decarboxylase 1 (mOdc1) in the FXP-ko cells. The C-term of mOdc1 is a well-known signal sequence for ubiquitin-independent proteasomal degradation [56–58]. While the mOdc1 peptide led to roughly 85–90% decreased expression of EGFP in control cells, this was very similar in all cell lines (Supplementary Fig. S7). Hence, ko of different FXPs has no general effect on proteasomal protein degradation.

**Fig. 3:**
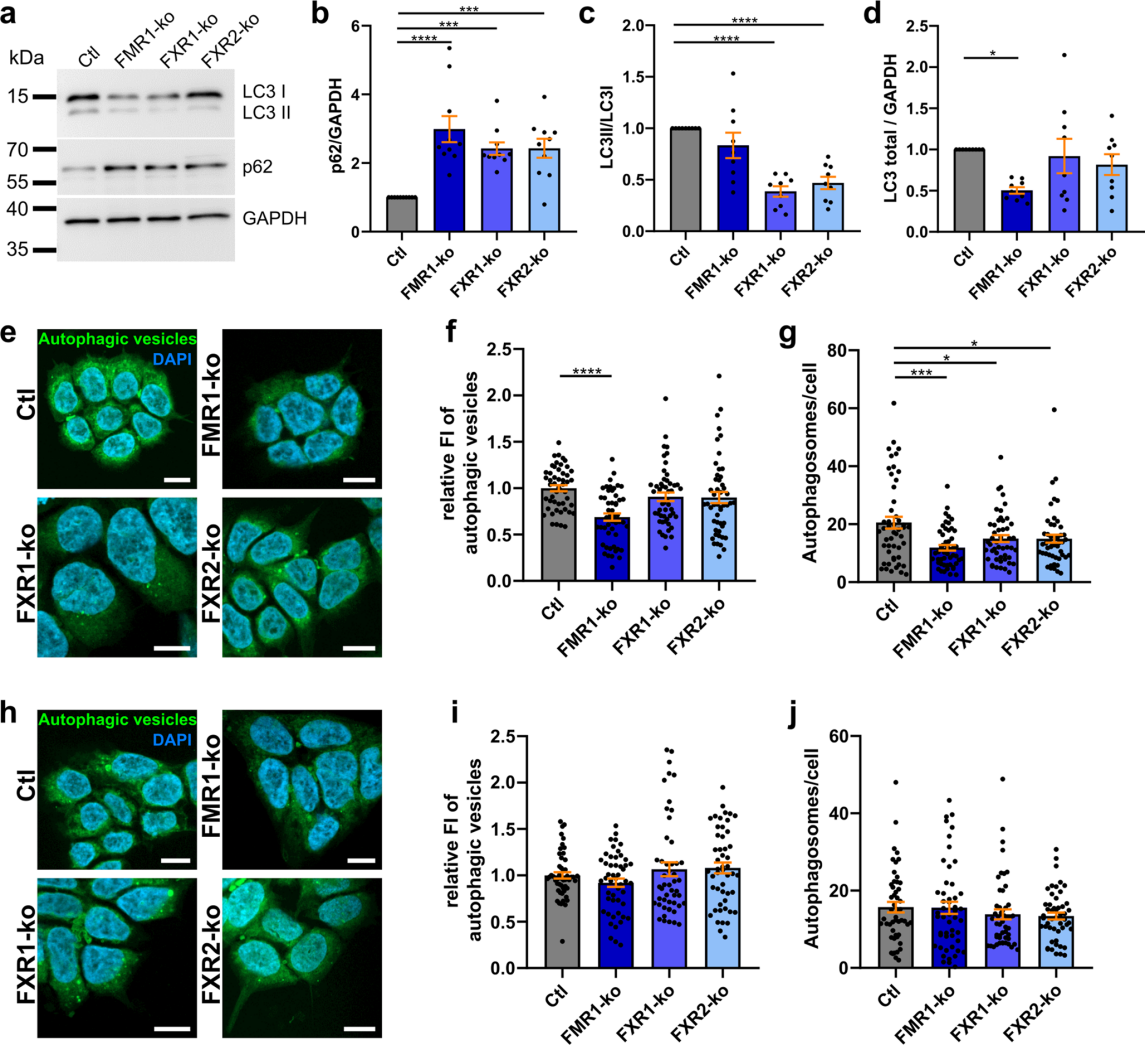
FXP deficiency impairs autophagy. **a-d** Western blot analyses of autophagy markers p62 (*SQSTM1*) and LC3 in HAP1 cells (**a**), and respective quantification of p62 (**b**), LC3II/I ratio (**c**), and total LC3 expression (**d**; *n* = 9–10). **e-j** Staining and quantification of fluorescence intensities and autophagic vesicle numbers in untreated (**e-g**) and rapamycin treated (500 nM for 18 h) cells (**h-j**) using a monodansylcadaverine-based dye largely overlapping with LC3 antibody staining (*n* = 70 images from 7 independent experiments; bars indicate mean ± SEM; *p < 0.05, **p < 0.01, ***p < 0.001, ****p < 0.0001 in a one-way ANOVA followed by *post hoc* Šídák’s test; scale bars are 10 μm; FI = fluorescence intensity)

Next, we addressed autophagy in the HAP1 cell lines. Using classical markers, Western blotting indicated defects in autophagy by accumulation of p62 (*SQSTM1*) in all FXP-ko cell lines while decreased LC3II/I ratios were detected in FXR1- and FXR2-ko cells only (Fig. 3a-c). However, despite unchanged LC3II/I ratio, accumulation of p62 and possibly reduced autophagy in FMR1-ko cells may be explained by decreased expression of total LC3 (Fig. 3d). We further validated these results by staining of autophagic vesicles in HAP1 cells using an optimized dye based on monodansylcadaverine that largely overlaps with LC3 antibody staining. Here, we found both decreased fluorescence intensities in FMR1-ko cells as well as decreased numbers of autophagic vesicles in all three FXP-ko cell lines (Fig. 3e-g). Thus, loss of individual FXPs is associated with defects in autophagy, but underlying mechanisms are different between ko of FMR1 and FXR1 or FXR2. Considering that impaired autophagy is a hallmark of most neurodegenerative diseases [59, 60], we validated this result in SH-SY5Y cells. Here, ≈ 50% kd of FXR1 or FXR2 led to both increased p62 intensities and puncta. This was not observed upon kd of FMR1 (Supplementary Fig. S8).

Loss of FMR1 has repeatedly been shown to activate AKT/mTOR pathway (e.g [61, 62])., and activated mTOR is well-known to inhibit autophagy [63]. Therefore, to test if increased mTOR signalling is involved in the defects of autophagy reported here, we treated FXP-ko cells with rapamycin. Indeed, mTOR inhibition rescued vesicle numbers in all FXP-ko cells, and staining intensity in FMR1-ko cells (Fig. 3h-j). Of note, experiments with rapamycin treated (Fig. 3h-j) and untreated (Fig. 3e-g) cells were performed in parallel, and respective data is directly comparable. Thus, overactivation of mTOR caused autophagy defects in FMR1-ko cells as expected, but also in FXR1- and FXR2-ko cells.

Since proteomics additionally indicated an increase of lysosomal components in the total and insoluble fraction of FXP-ko cells, we used Lysotracker to visualize lysosomes, and found increased Lysotracker signal exclusively in FMR1-ko cells (Supplementary Fig. S9a, b). As lysosomal dysfunction is closely associated with various neurodegenerative diseases [64–66], we additionally performed Lysotracker staining in SH-SY5Y cells upon knockdown of the FXPs. Here, we found increased Lysotracker signal upon kd of FXR2 only (Supplementary Fig. S9c, d). To further characterize accumulation of lysosomes in HAP1 cells, we examined possible damage of lysosomes by determining correlation of fluorescence signals of lysosomal marker LAMP2 and GAL8, a marker of damaged membranes [67–69]. Surprisingly, co-localization analysis did not indicate an increase but a decrease of damaged lysosomes in all FXP-ko cell lines. Notably, large fluctuations in LAMP2/GAL8 co-localization were observed exclusively in FMR1-ko cells (Supplementary Fig. S10a, b). Additionally, we noted that lysosomal marker LAMP2, in contrast to Lysotracker signal, was not increased in FMR1-ko cells (Supplementary Fig. S10c, d). Re-analysis of Lysotracker staining in HAP1 and SH-SY5Y cells, i.e. determination of numbers of stained vesicles and the area covered by lysosomes, confirmed initial analyses of fluorescence intensities (Supplementary Fig. S10e-h). As Lysotracker is not specific for lysosomes but stains acidic organelles in general, we additionally stained early and late endosomes using markers RAB5 and RAB7, respectively. While early endosomes were unchanged in the FXP-ko cells, we detected a decrease of late endosomes in the FMR1-ko cells (Supplementary Fig. S11). Therefore, we hypothesize that loss of FMR1/FXR2 induces changes in the maturation of late endosomes to lysosomes that may include premature acidification of endosomes and/or timing of specific proteins incorporated/removed. However, more detailed analyses are required to explain these observations.

### Loss of FMR1 leads to accumulation of damaged mitochondria while FXR1 and FXR2 may function in mitochondrial fission

**Fig. 4:**
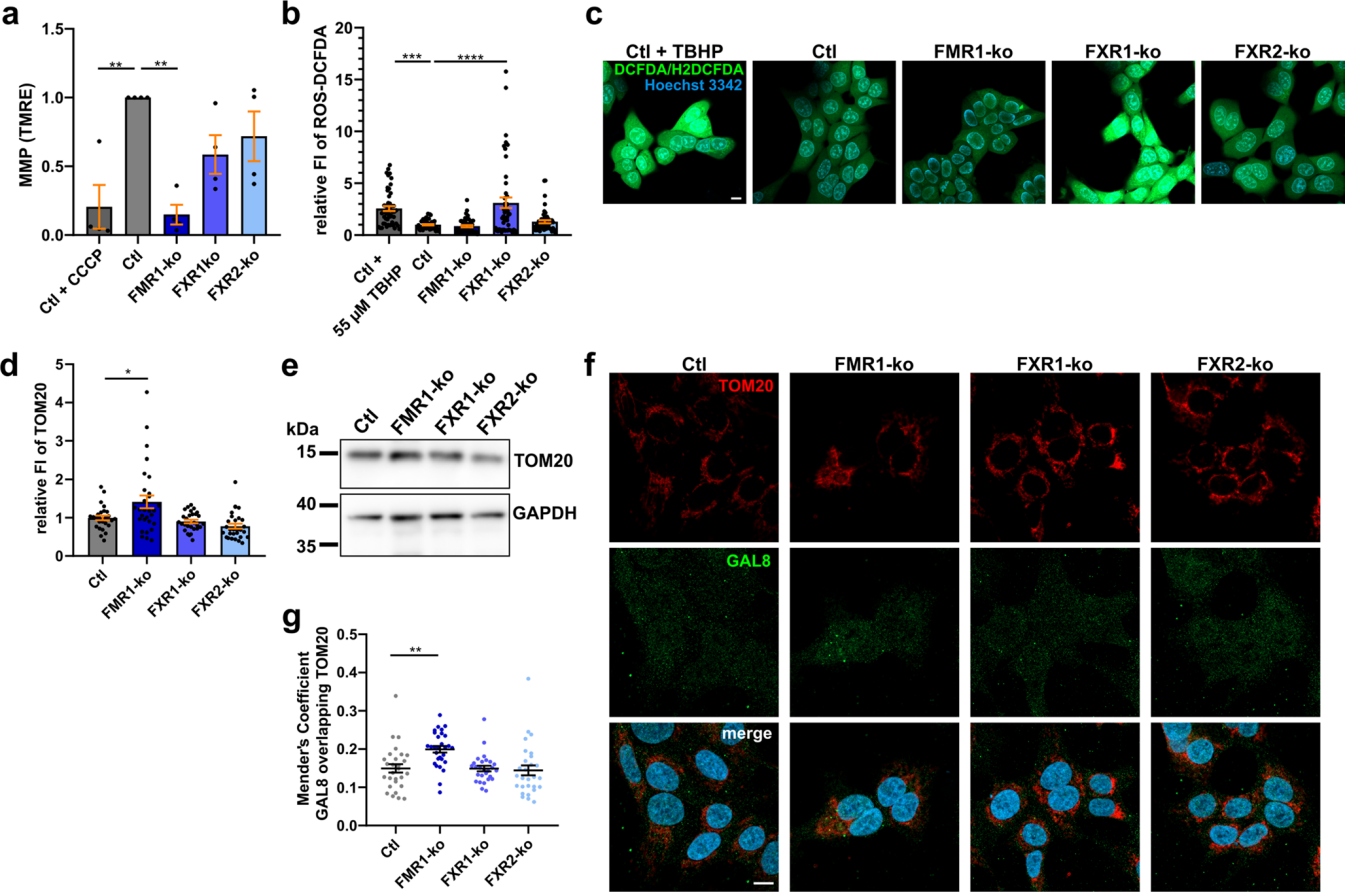
Loss of FMR1 impairs mitochondrial health in HAP1 cells. **a** TMRE-based assessment of mitochondrial membrane potential in HAP1 cells by FACS analyses. Control cells treated with CCCP are shown as positive control (*n* = 4). **b**,** c** Quantification of fluorescence intensities of the ROS detecting dye DCFDA (**b**) and respective representative images (**c**). Control cells treated with ROS inducer TBHP (55 µM for 45 min) are shown as positive control (*n* = 50 images from 5 independent experiments). **d**,** e** Relative fluorescence intensities (**d**) and Western blot (**e**) of mitochondrial membrane marker TOM20. **f** Representative images of the quantifications shown in (**d**) and (**g**). **g** Mender’s coefficient of GAL8 overlapping TOM20 (*n* = 29 images from 3 independent experiments; bars indicate mean ± SEM; *p < 0.05, **p < 0.01, ***p < 0.001, ****p < 0.0001 in a one-way ANOVA followed by *post hoc* Šídák’s test; scale bars are 10 μm; MMP = mitochondrial membrane potential, FI = fluorescence intensity)

Considering that proteomics revealed various metabolic changes induced by the loss of individual FXPs (Fig. 1c), and because the KEGG term “Ribosome” enriched in downregulated proteins of the total and insoluble proteome of FXR2-ko cells is mostly driven by mitochondrial ribosomal proteins (Supplementary Table S7 + S8), we decided to further examine consequences of FXP loss on mitochondria. First, we used TMRE to detect possible changes in mitochondrial membrane potentials using both fluorescence microscopy (Supplementary Fig. S12) and FACS analyses (Fig. 4a). In both cases, we detected severely reduced membrane potentials after ko of FMR1. Since mitochondrial dysfunction is a hallmark of most neurodegenerative diseases [59, 60], we additionally examined SH-SY5Y cells for mitochondrial dysfunction upon FXP kd. Here, reporter protein mCherry used for the identification of transfected cells prevented the use of TMRE. Instead, we used JC-1 dye emitting green fluorescence when excluded from unhealthy mitochondria (low membrane potential), and red fluorescence when entering healthy mitochondria. Due to mCherry, we could evaluate the green channel only. Nevertheless, increased green fluorescence upon kd of FMR1 indicated similar changes as observed in HAP1 cells (Supplementary Fig. S13). Decreased membrane potential in FMR1-ko HAP1 cells was neither accompanied by decreased ATP level (Supplementary Fig. S14a) nor by increased production of reactive oxygen species (ROS). Surprisingly, loss of FXR1 strongly induced ROS production (Fig. 4b, c). Additionally, we tested mitochondrial motility by video tracking and mitophagy by expression of mitophagy sensor mt-Keima, a fluorescent protein excited at shorter wavelengths (440 nm) when in neutral pH of mitochondria, and at longer wavelengths (586 nm) when in the acidic environment of lysosomes upon mitophagy [70, 71]. However, in both cases we did not detect any differences between the cell lines (Supplementary Fig. S14b-d). As mitochondrial defects in FMR1-ko cells were not accompanied by increased mitophagy, we hypothesized that damaged mitochondria may accumulate in these cells. Indeed, increased fluorescence intensities and Western blot signals of mitochondrial membrane marker TOM20 (Fig. 4d-f), and increased co-localization of TOM20 and GAL8 (Fig. 4f, g) exclusively in FMR1-ko cells support this hypothesis.

Of note, we additionally performed ICC and Western blots of mitochondrial matrix marker HSP60 leading to different results compared to TOM20. Here, FXR1- and FXR2-, but not FMR1-ko cells showed increased HSP60 intensities (Supplementary Fig. S15a-c). This imbalance in mitochondrial membrane and matrix markers may reflect differences in surface/volume ratios, and may be explained by mitochondrial fission/fusion events. FMR1 is involved in mitochondrial fission by regulating local translation of mitochondrial fission factor (MFF; [72]). When testing co-localization of streptavidin-labelled mitochondria and MFF, we found decreased co-localization in FXR1- and FXR2-, but not FMR1-ko cells (Supplementary Fig. S15d, e). Overall MFF expression was unchanged in all FXP-ko cell lines (Supplementary Fig. S15f, g). These findings may explain imbalances of mitochondrial markers, and involve FXR1 and FXR2 in mitochondrial fission/fusion dynamics.

###  Loss of individual FXPs induces a senescence-like phenotype

**Fig. 5:**
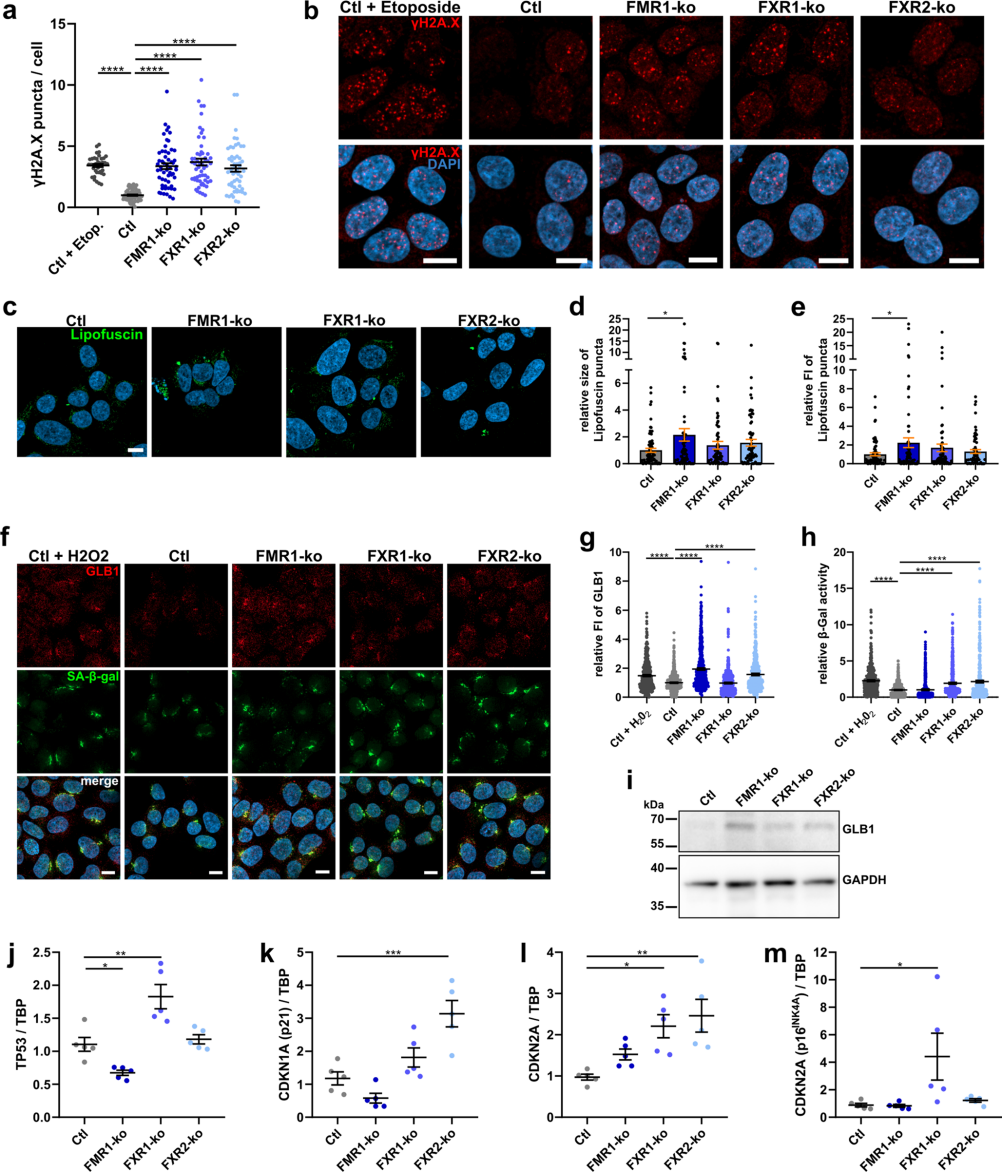
Loss of FXPs induces senescence-like phenotypes. **a** Numbers of γH2A.X puncta in HAP1 cells. Control cells treated with DNA damage inducer etoposide (1 µM for 30 min) are shown as positive control (*n* = 36–54 images from 3–5 independent experiments). **b** Representative images of the quantification shown in (**a**). **c-e** Representative images (**c**) and quantification of relative size (**d**) and fluorescence intensity (**e**) of autofluorescent lipofuscin puncta (*n* = 74 images from 7 independent experiments). **f-h** Representative images (**f**) and quantification of ß-Galactosidase expression (GLB1; **g**) and activity (SA-ß-gal; **h**) in HAP1 cells. Control (Ctl) cells treated with H_2_O_2_ (200 µM for 2 h) are shown as positive control for increased SA-ß-gal activity (*n* = 90–120 cells from 3 independent experiments). **i** Western blot of ß-Galactosidase (GLB1) in HAP1 cells. **j-m** Measurement of relative abundances of mRNAs coding for p53 (*TP53*; **j**), p21 (*CDKN1A*; **k**), CDKN2A (host gene of p16^INK4A^; **l**) and p16^INK4A^ (senescence-associated splice variant of *CDKN2A*; **m**). Data were normalized to TBP expression (*n* = 6; bars indicate mean ± SEM; *p < 0.05, **p < 0.01, ***p < 0.001, ****p < 0.0001 in a one-way ANOVA followed by *post hoc* Šídák’s test; scale bars are 10 μm; FI = fluorescence intensity; TBP = TATA-box binding protein)

Cellular senescence was among the top enriched KEGG terms in our proteomic data, and is involved in cancer [73, 74] as well as in neurodegenerative diseases [75–77]. Therefore, we examined some hallmarks of cellular senescence [78–80] in the FXP-ko HAP1 and FXP-kd SH-SY5Y cells. In both cell lines loss of each FXP led to increased DNA damage measured by numbers of nuclear puncta of DNA damage marker γH2A.X (Fig. 5a, b; Supplementary Fig. S16). Accumulation of lipofuscin, however, was restricted to the loss of FMR1 in both HAP1 and SH-SY5Y cells (Fig. 5c-e; Supplementary Fig. S17). The senescence-associated increase in ß-Galactosidase (GLB1) activity is likely a mixture of both increased activity and expression [81]. Therefore, we measured both in our cell lines. In HAP1 cells, loss of FMR1 and FXR2 induced increased expression of GLB1 while increased activity was evident upon loss of FXR1 and FXR2 (Fig. 5f-i). In contrast, in SH-SY5Y cells, we found no increase of GLB1 expression upon kd of the FXPs, and increased activity was restricted to the kd of FMR1 (Supplementary Fig. S18).

Generally, pathways leading to cellular senescence involve increased expression of either p21 (*CDKN1A*) or p16^INK4A^, or both. While p21 may be induced by increased/activated p53 (*TP53*), senescence signals may also directly induce expression of p16^INK4A^ which is a splice variant of the host gene *CDKN2A* [82–84]. To determine which pathway may be activated upon loss of individual FXPs, we measured expression of *TP53* and *CDKN1A*, as well as of host gene *CDKN2A* and the splice variant p16^INK4A^ in the HAP1 FXP-ko cell lines by qRT-PCR (Fig. 5j-m). In FXR1-ko cells, increased expression of all mRNAs measured indicates activation of both pathways, but the increase in p21 (*CDKN1A*) did not reach statistical significance. In FXR2-ko cells we found strong induction of p21 (*CDKN1A*), but, surprisingly, without induction of p53 (*TP53*). Additionally, the host gene of p16^INK4A^ was induced, but not the senescence-associated splice variant p16^INK4A^. Interestingly, in FMR1-ko cells we could not detect induction of any of these genes, and expression of p53 (*TP53*) was even reduced. Hence, loss of FXR1 and FXR2 differentially activates cascades leading to cellular senescence, while FMR1 likely induces senescence by alternative mechanisms. Table 1 provides an overview of all findings from HAP1 FXP-ko and SH-SY5Y FXP-kd cells presented in this study.

**Table 1:** Summary of results derived from FXP-ko HAP1 and FXP-kd SH-SY5Y cells

	HAP1 knockout			SH-SY5Y knockdown		
	FMR1	FXR1	FXR2	FMR1	FXR1	FXR2
Ribosome biogenesis						
Mature ribosome abundance	**↔**	↔	↔	n.d.	n.d.	n.d.
Size of nucleoli	↔	↑	↑	n.d.	n.d.	n.d.
47 S rRNA abundance	↔	↔	↓	n.d.	n.d.	n.d.
18 S rRNA abundance	↔	↑	↑	n.d.	n.d.	n.d.
***Protein aggregation***						
Proteostat intensity	↑	↔	↔	n.d.	n.d.	n.d.
Aggregation of proteostasis sensors	↔	↔	↔	n.d.	n.d.	n.d.
Translational fidelity	↔	↔	↓	n.d.	n.d.	n.d.
Misfolded proteins	↓	↓	↔	n.d.	n.d.	n.d.
Protein synthesis	↑	↔	↔	n.d.	n.d.	n.d.
TDP-43 solubility upon stress	↔	↔	↔	n.d.	n.d.	n.d.
***Protein degradation***						
Proteasomal activity	↔	↔	↔	n.d.	n.d.	n.d.
Global protein ubiquitination	↔	↔	↔	n.d.	n.d.	n.d.
Ubiquitin-independent degradation	↔	↔	↔	n.d.	n.d.	n.d.
p62 (*SQSTM1*) level	↑	↑	↑	↔	↑	↑
LC3II/LC3I ratio	↔	↓	↓	n.d.	n.d.	n.d.
Total LC3 expression	↓	↔	↔	n.d.	n.d.	n.d.
Autophagic vesicles	↓	↓	↓	n.d.	n.d.	n.d.
Lysotracker intensity	↑	↔	↔	↔	↔	↑
Lysosomes (LAMP2)	↔	↔	↔	n.d.	n.d.	n.d.
Damaged lysosomes	↓	↓	↓	n.d.	n.d.	n.d.
Early endosomes (RAB5)	↔	↔	↔	n.d.	n.d.	n.d.
Late endosomes (RAB7)	↓	↔	↔	n.d.	n.d.	n.d.
***Mitochondria***						
Membrane potential	↓	↔	↔	↓	↔	↔
ATP level	↔	↔	↔	n.d.	n.d.	n.d.
Reactive oxygen species (ROS)	↔	↑	↔	n.d.	n.d.	n.d.
Mitophagy	↔	↔	↔	n.d.	n.d.	n.d.
Mitochondrial motility	↔	↔	↔	n.d.	n.d.	n.d.
Damaged mitochondria	↑	↔	↔	n.d.	n.d.	n.d.
Mitochondrial fission	↔	↓	↓	n.d.	n.d.	n.d.
***Cellular senescence***						
DNA damage	↑	↑	↑	↑	↑	↑
Lipofuscin	↑	↔	↔	↑	↔	↔
ß-Galactosidase expression	↑	↔	↑	↔	↔	↔
ß-Galactosidase activity	↔	↑	↑	↑	↔	↔
p53 (*TP53*) mRNA	↓	↑	↔	n.d.	n.d.	n.d.
p21 (*CDKN1A*) mRNA	↔	↔	↑	n.d.	n.d.	n.d.
p16^INK4A^ host gene (*CDKN2A*) mRNA	↔	↑	↑	n.d.	n.d.	n.d.
p16^INK4A^ (splice variant *CDKN2A*) mRNA	↔	↑	↔	n.d.	n.d.	n.d.

↔ = unchanged; ↑ = increased; ↓ = decreased; n.d. = not determined;

## Discussion

In this study, we comprehensively analysed cellular defects associated with the loss of FXR1 and FXR2, respectively, and compared results to the loss of well-studied FMR1. Our unbiased proteomic screen revealed implication of this protein family in basic cellular processes, such as ribosome biogenesis, autophagy and endo-lysosomal pathways, mitochondrial health and dynamics, and especially in cellular senescence. More detailed analyses confirmed most of the proteomic findings, but simultaneously revealed substantial differences associated with the loss of individual FXPs. Overall, our study provides novel insights into various diseases implying dysregulation of members of this multifunctional protein family.

The initial proteome analysis of HAP1 cells indicated shared and unique functions of the individual FXPs, and emphasises the importance of these proteins in basic cellular processes. Previous proteomic studies were largely restricted to neuronal cells or tissues of mice and humans suffering from FXS, and mostly focused on dominating synaptic changes. A recent study [85] used an alternative proteomic approach to identify DEPs in FMR1-ko SH-SY5Y cells, and results are very similar to results from FXP-ko HAP1 cells when proteomic analyses are performed analogous to ours. However, up- and downregulated proteins (*p*_*adjusted*_ < 0.05) were not enriched in proteins contributing to KEGG pathways “Ubiquitin mediated proteolysis”, “Ribosome biogenesis in eukaryotes” and “Cellular senescence”, respectively. Most likely, this is because significantly less proteins were quantified in this study (3358 vs. 4656), and the vast majority of proteins driving these terms in HAP1 cells were not detected. Hence, direct comparisons are difficult here. Nevertheless, previous studies already linked loss of FMR1 to metabolic changes and mitochondrial dysfunction, ribosomes as well as protein folding [86]. Here, we could confirm and extend previous findings, and directly compare loss of FMR1 to the loss of FXR1 and FXR2 which have not been studied on a proteome-wide scale yet.

The most striking finding of this study is the strong link of all three FXPs to cellular senescence important for both neurodegeneration and cancer. While FXR1 was already linked to senescence induction in smooth muscle cells [87] and senescence evasion in specific types of cancer [22, 24–27], our study suggests similar functions of FMR1 and FXR2, but by different pathways. Actually, also other FXP-related mechanisms reported here, such as decreased autophagy and mitochondrial dysfunction, may not be included in the hallmarks of cellular senescence [78–80], but of organismal aging [88, 89]. Similarly, defects in ribosome biogenesis including enlarged nucleoli and accumulation of 18 S rRNA are strongly associated with (premature) aging and longevity [90–92], but are not included in hallmarks of aging/senescence yet. While in neurodegenerative diseases accumulation of senescent cells in the CNS contributes to neurodegenerative phenotypes [75–77], senescence evasion is involved in tumorgenesis and cancer progression [73, 74, 93, 94]. Our data indicate that all three FXPs play an important role in cellular pathways leading to senescence, and loss of a single FXP family member is sufficient to induce a senescence-like phenotype, i.e. to induce some but not all hallmarks of senescence. Importantly, despite high homology, pathways leading to senescence are different for each FXP in the same cell type. Surprisingly, at least at the RNA level, we could not measure any induction of either p21 (*CDKN1A*) or p16^INK4A^ (splice variant of *CDKN2A*) in FMR1-ko cells. However, mRNAs of p16^INK4A^ targets CDK4 and CDK6 have been reported to directly interact with FMR1, but not with FXR1 or FXR2 [95]. Hence, senescence induction by loss of FMR1 may occur independently of p16^INK4A^ by causing dysregulation of CDK4/6 expression, but this hypothesis requires verification. Nonetheless, our results suggest a close relation of all three FXPs to cellular senescence. Interestingly, despite limited data available, premature aging is evident in FXS patients [96–98], and an age-dependent decline of FMR1 in brains of physiologically aging rats has been reported [99], further supporting the strong link of at least FMR1 to cellular senescence in vivo.

Another important finding of our study is the high cell type specificity of defects induced by loss of single FXPs. For example, defects in ribosome biogenesis including enlarged nucleoli assessed by Fibrillarin were reported before in hippocampal neurons of FMR1-ko mice [48], but were absent in FMR1-ko HAP1 cells. Here, comparable defects in ribosome biogenesis were restricted to FXR1- and FXR2-ko cells. Similarly, decreased mitochondrial membrane potentials were reported in primary cortical neurons from FMR1-ko mice [100], but not in colorectal adenocarcinoma cell line DLD-1 upon knockdown of FMR1 [101]. Here, in HAP1 and SH-SY5Y cells, only loss of FMR1 induced depolarization of mitochondria, but impaired mitochondrial fission reported in primary neurons of mice and rats upon loss of FMR1 [72] was restricted to FXR1- and FXR2-ko HAP1 cells. Therefore, consequences associated with the loss of a specific FXP are highly dependent on the respective cell type, and may vary depending on FXP expression level, compensatory capacities and/or other factors expressed.

In neurodegenerative diseases, loss of FXP expression has been repeatedly correlated with pathogenic protein aggregation [3], but evidence for direct involvement of the FXPs is lacking so far. Our analyses of insoluble FXP-ko HAP1 proteomes did not indicate increased aggregation/insolubility of proteins including some forming intracellular inclusions in neurodegenerative diseases. Additionally, our cell biological assays could not detect generally increased protein aggregation. The only exception here were increased fluorescence intensities of Proteostat in FMR1-ko cells. As translation was increased exclusively in FMR1-ko cells, this may be due to increased protein load in these cells, or, alternatively, represent very early stages of accumulating, but not misfolded, proteins that are not stable upon cell lysis by sonication. Furthermore, when cells were stressed with sodium arsenite, loss of individual FXPs had no effect on the solubility of TDP-43. Therefore, it is rather unlikely that the solubility of aggregation-prone proteins is modulated by protein-protein interactions with FXPs, as it has been suggested for FMR1 and TDP-43 [15]. Our data support an alternative model, in which other mechanisms related to the FXPs, such as impaired autophagy, mitochondrial dysfunction, increased DNA damage, and/or cellular senescence contribute to neurodegenerative phenotypes. It is noteworthy that FXP functions reported here and elsewhere largely overlap with converging disease mechanisms evident in most neurodegenerative diseases [59, 60]. So far, it is unknown if defects in such mechanisms are indeed caused by FXP loss. Nonetheless, overexpression of (d)FMR1 substantially rescued the phenotypes at least in ALS animal models [13, 15]. While in both models, molecular analyses were largely restricted to neuromuscular junctions, it is likely that other mechanisms were rescued as well. At least FMR1 is closely linked to cellular stress responses, whereby higher and lower expression are mostly associated with increased and decreased cell viability, respectively [102–104]. Consequently, regardless if FXP loss directly impairs pathways leading to neurodegeneration, restoring FXP expression may improve important disease-associated pathways, and may have beneficial effects for patients suffering from various neurodegenerative diseases.

In most cancers at least one of the FXPs is upregulated, and in some FXP expression level are even of prognostic value [17, 18]. Although we used a FXP knockout/knockdown paradigm here, some of our findings may be inversely regulated upon overexpression of these proteins. Interestingly, cancer cells of diverse origins activate pathways usually operative in neurons, which are linked to FMR1 [20, 105, 106]. Given inverse regulation in cancer, our data may at least partially explain increased fitness of cancer cells, and contribute to better understanding the role of the FXPs.

While our study indicates strong links of all three FXPs to cellular senescence and aging, it also has some limitations. First, we focused on KEGG pathways only for validation of proteomic findings, and disregarded alternative databases. Second, we could not elucidate all molecular details and mechanisms associated with the loss of individual FXPs. Especially the relation of these proteins to lysosomes/endosomes and mitochondria require further investigation. Third, we used a knockout/knockdown paradigm in cell lines here, and it is not clear if overexpression of these proteins in cancer leads to opposite effects, or if results from SH-SY5Y cells are transferable to postmitotic neurons without restrictions. Forth, we studied loss of single FXPs, but in cancer [17, 18] as well as in neurodegenerative diseases [14] abundance of two or all three FXPs may be increased or decreased simultaneously, respectively.

Overall, our unbiased proteomic study indicates that modulation of cellular senescence and aging may represent another important function of this protein family beyond regulation of local translation in neurons. Our data provides a valuable resource and starting points for further research on this protein family in respective cell types affected by age-related diseases, and in physiological aging in general.

## Supplementary Information

Below is the link to the electronic supplementary material.

Supplementary Material 1 (XLSX. 1.65 MB)

Supplementary Material 2 (XLSX. 1.60 MB)

Supplementary Material 3 (PDF. 2.79 MB)

## Data Availability

The datasets generated and/or analysed during this study are available within the article and its Supplementary Information files.

## Abbreviations

AD: Alzheimer’s disease
ALS: Amyotrophic Lateral Sclerosis
Aß: Amyloid-beta
CCCP: Carbonyl Cyanide m-Chlorophenylhydrazone
Ctl: Control
DEP: Differentially expressed protein
FACS: Fluorescence activated cell sorting
FI: Fluorescence intensity
Fluc: Firefly luciferase
FlucDM: Fluc double mutant
FlucSM: Fluc single mutant
FXP: Fragile X protein
FXPOI: Fragile X–associated premature ovarian insufficiency
FXS: Fragile X syndrome
FXTAS: Fragile X–associated tremor/ataxia syndrome
ICC: Immunocytochemistry
kd: knockdown
ko: knockout
LFQ: Label-free quantification
MFF: Mitochondrial fission factor
MMP: Mitochondrial membrane potential
mOdc1: mouse Ornithine decarboxylase 1
NanoLuc: Nanoluciferase
PCA: Principal component analysis
PD: Parkinson's disease
ROS: Reactive oxygen species
shScr: shRNA scrambled
TBP: TATA-box binding protein
TCEP: Tris(2-carboxyethyl)phosphine hydrochloride
TEAB: Triethylammonium bicarbonate
TMRE: Tetramethylrhodamine ethyl ester, perchlorate
TPE-MI: Tetraphenylethene maleimide
UTR: Untranslated region
