# Supplementary material for: Comparing loss of individual fragile X proteins suggests strong links to cellular senescence and aging
Source: Cell Mol Life Sci. 2025 Oct 21;82(1):358. doi: 10.1007/s00018-025-05898-0 (PMC12540212; doi:10.1007/s00018-025-05898-0)
Supplement: Supplementary file 3 — Supplementary Material 3 (PDF. 2.79 MB) [file 18_2025_5898_MOESM3_ESM.pdf]

Comparing Loss of Individual Fragile X Proteins Suggests Strong Links to Cellular Senescence and Aging

Sonja Menge[1], Inmaculada Segura[2,3], Max Hartmann[4], Lorena Decker[1], Selin Kiran[1], Karin M. Danzer[1,5], Sebastian Iben[4], Angelika B. Harbauer[2,6,7], Patrick Oeckl[1,5], Axel Freischmidt[1,#]

[1]Department of Neurology, Ulm University, Ulm, Germany

[2]Max Planck Institute for Biological Intelligence, Martinsried, Germany

[3]Biomedical Center Munich, Department of Cellular Physiology, Ludwig-Maximillian-University, Martinsried, Germany

[4]Department of Dermatology and Allergic Diseases, Ulm University, Ulm, Germany

[5]German Center For Neurodegenerative Diseases (DZNE) Ulm, Ulm, Germany

[6]Technical University of Munich, School of Medicine and Health, Institute of Neuronal Cell Biology, Munich, Germany

[7]Munich Cluster for Systems Neurology, Munich, Germany

[#]Corresponding author: Axel Freischmidt
Ulm University
Department of Neurology, ZBMF,
Helmholtzstr. 8/1
89081 Ulm
Germany
Tel: +49 731 500 44706
email: axel.freischmidt@uni-ulm.de

Supplementary Material

Supplementary Figures

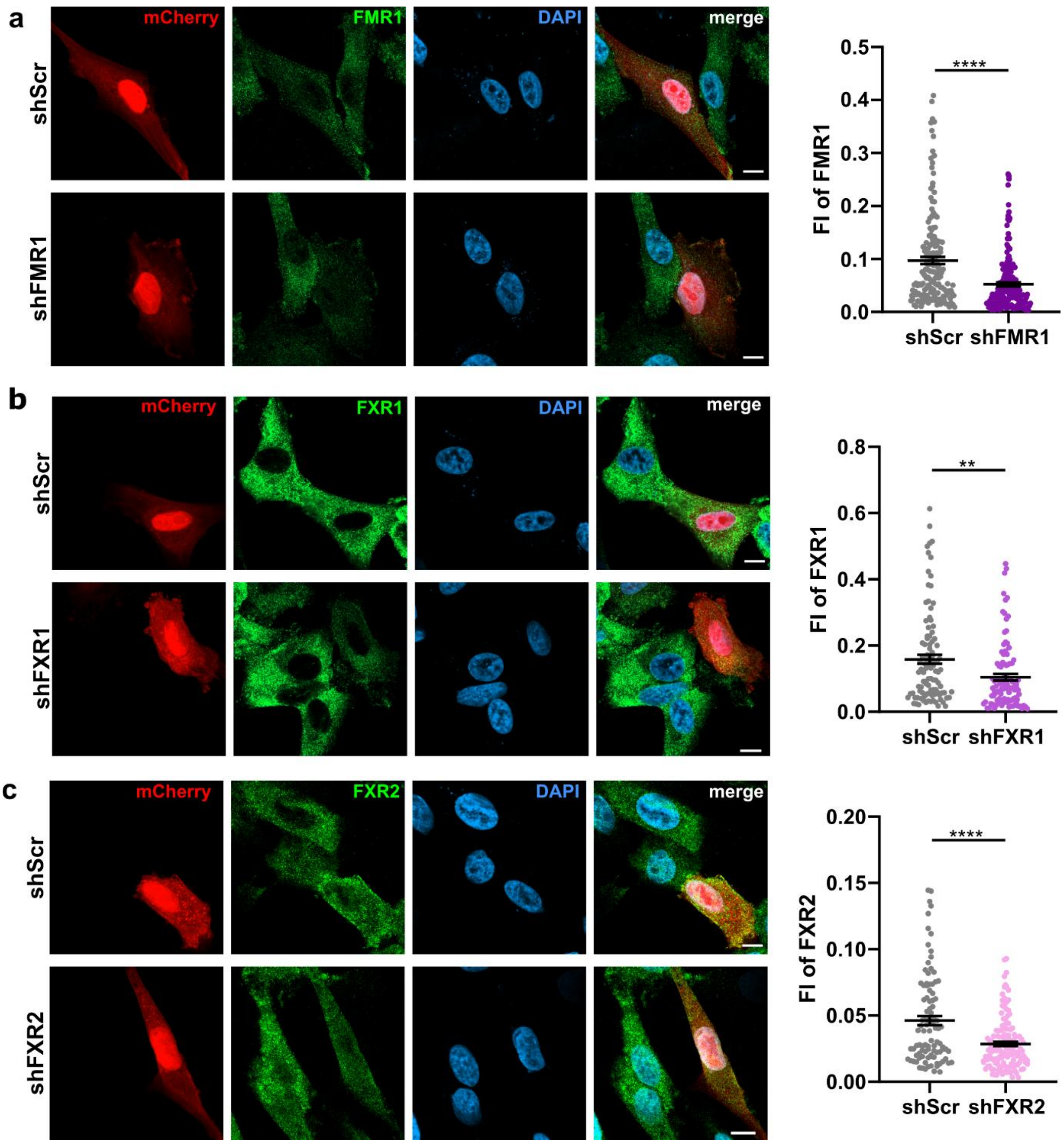

Supplementary Fig. S1 shRNA mediated knockdown of FXPs in SH-SY5Y cells. **a-c** SH-SY5Y cells transfected with shRNA targeting FMR1 (**a**), FXR1 (**b**), or FXR2 (**c**) were analysed 72 h post transfection using respective antibodies against each FXP. mCherry encoded in the same plasmid was used to identify transfected cells and for normalizing transfection efficiencies (n = 100 - 150 transfected cells from 4 independent experiments; bars indicate mean ± SEM; ****p*<0.01, *******p*<0.0001 in an unpaired, two-tailed Student's *t*-test; scale bars are 10 µm; FI = fluorescence intensity).

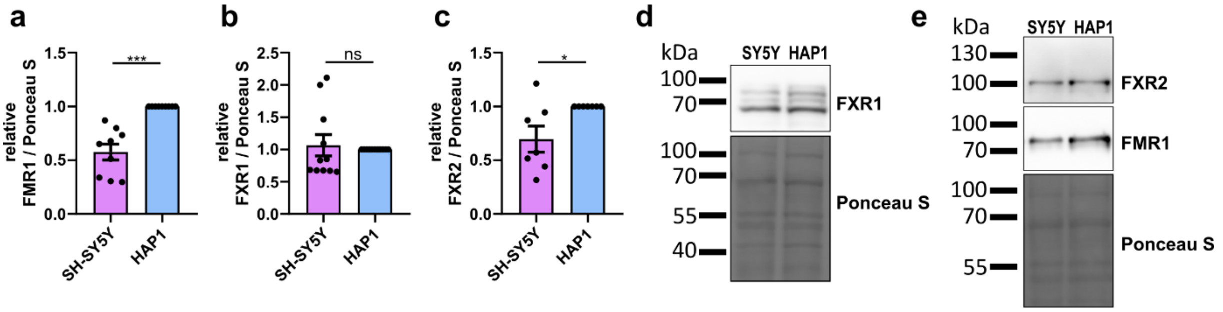

Supplementary Fig. S2 Basal FXP protein level in SH-SY5Y and HAP1 cells. **a-c** Quantification of Western blots comparing expression of FMR1 (**a**), FXR1 (**b**) and FXR2 (**c**) in untreated SH-SY5Y cells and HAP1 control cells. Band intensities were normalized to total protein (Ponceau S). **d, e** Representative images of Western blots (n = 9 - 11; bars indicate mean ± SEM; *p<0.05; ***p<0.001 in an unpaired, two-tailed Student's t-test).

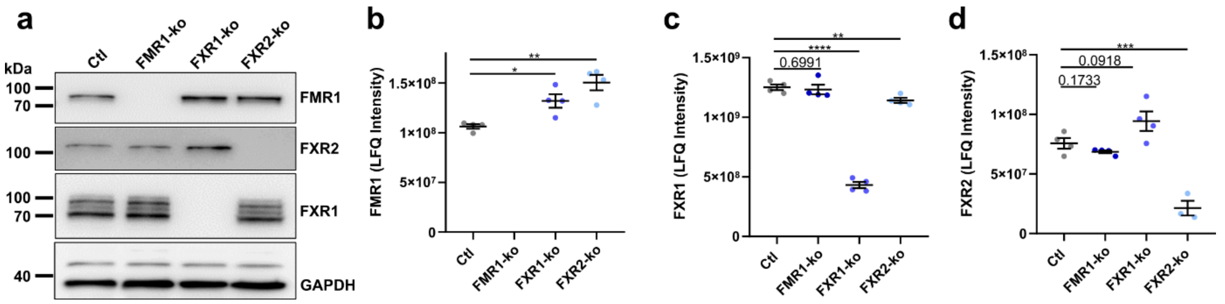

Supplementary Fig. S3 FXP expression in HAP1 control and FXP-ko cells. **a** Western blot comparing expression of FMR1, FXR1 and FXR2 in HAP1 control and FXP-ko cell lines. **b-d** Label-free quantification (LFQ) intensities of FMR1 (**b**), FXR1 (**c**) and FXR2 (**d**) in HAP1 cells as determined by proteomics (n = 4; bars indicate mean ± SEM; *p<0.05; **p<0.01; ***p<0.001; ****p<0.0001 in an unpaired, two-tailed Student's t-test).

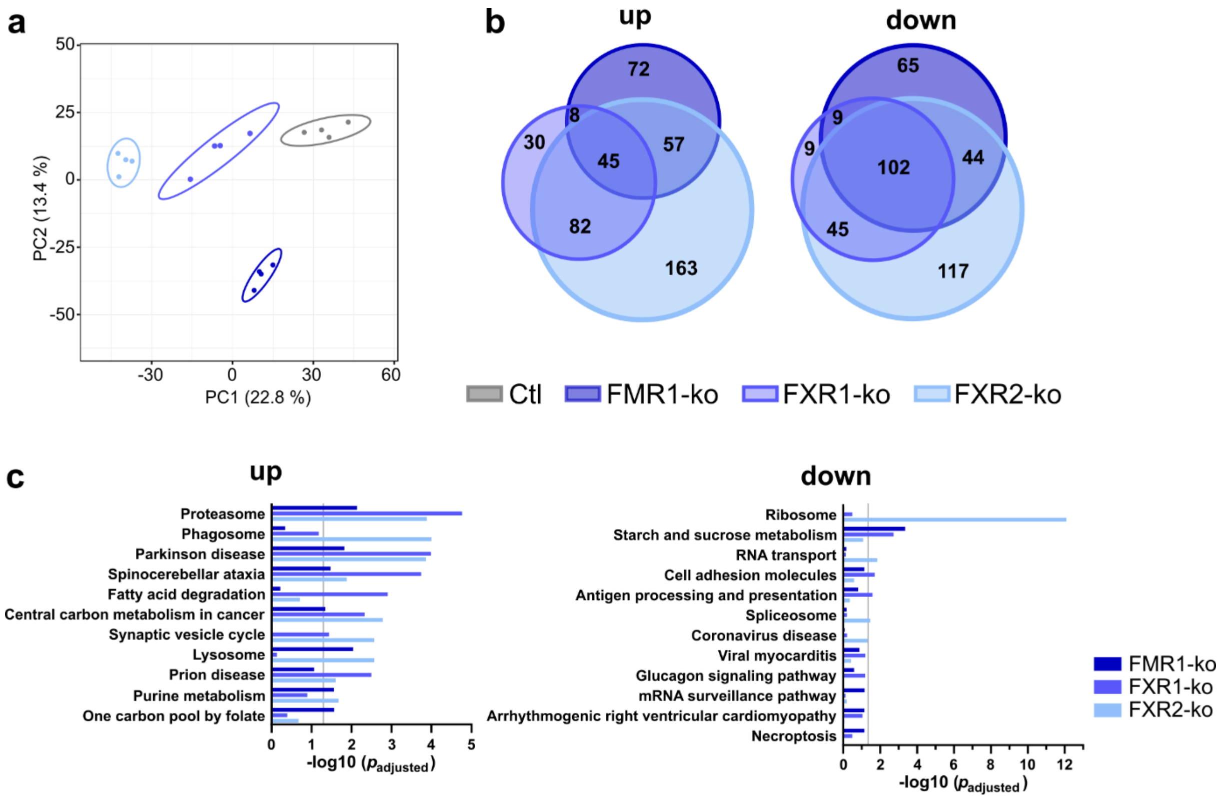

Supplementary Fig. S4 Loss of individual FXPs leads to increased components of the proteasome and autophagic/lysosomal vesicles in the insoluble proteome. **a** Principal component analyses (PCA) of 3802 proteins reliably quantified in the insoluble proteome of HAP1 control (Ctl) and FXP-knockout (FXP-ko) cells. **b** Overlap of robustly up- and downregulated proteins in the insoluble fraction of HAP1 cell lines. **c** KEGG pathway enrichment analyses of proteins with increased and decreased abundance, respectively. Top 5 KEGG pathways for each FXP including results for the other FXPs in the respective pathway are shown. Vertical lines indicate the significance threshold ($p_{adjusted}$ = 0.05; n = 4 for each cell line).

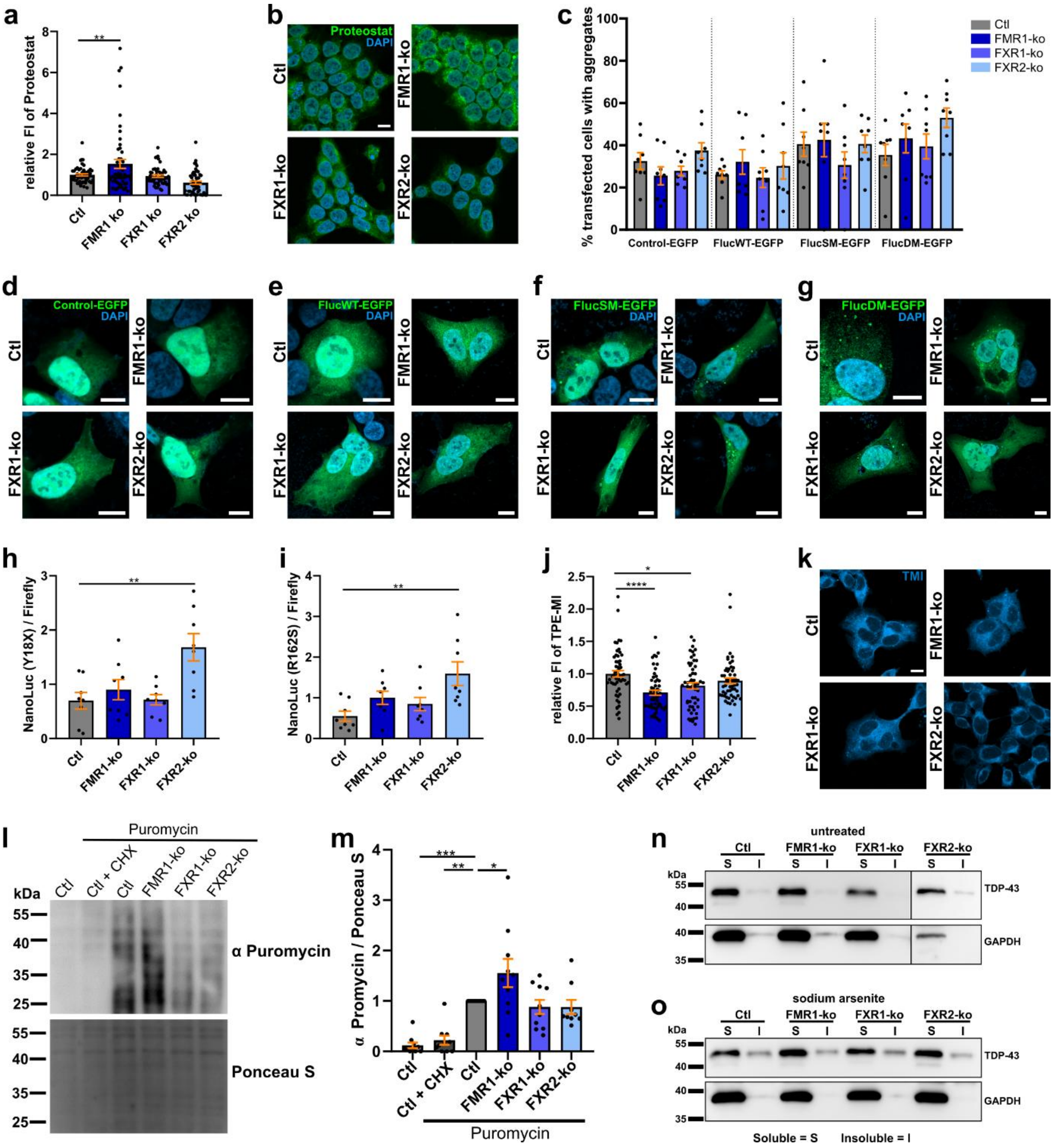

Supplementary Fig. S5 FXP loss does not alter protein aggregation and folding. **a, b** Quantification of fluorescence intensities (**a**) and representative images (**b**) of unspecific staining of protein aggregates and/or oligomeric protein assemblies (aggresomes) using the PROTEOSTAT® Aggresome detection kit in FXP-ko cells (n = 50 images from 5 independent experiments). **c-g** Quantification of cells containing aggregates (**c**) and representative images of HAP1 Ctl and FXP-ko cells transfected with plasmids encoding EGFP (**d**), Fluc-EGFP (**e**), FlucSM-EGFP (**f**) or FlucDM-EGFP (**g**) 48 h post transfection (n = 8, 10 - 11 images each). **h, i** Luminescence of NanoLuc (Y18X; **h**) and NanoLuc (R162S; **i**) expressed for 24 h in HAP1

cells. Luminescence of co-transfected firefly luciferase was used to normalize for different transfection efficiencies (n = 8, triplicates measured each). **j, k** Quantification of fluorescence intensities (**j**) and representative images (**k**) of HAP1 cell lines stained with TPE-MI dye (n = 66 images from 5 independent experiments). **l, m** Representative image (**l**) and quantification (**m**) of Western blots of HAP1 cells treated with puromycin (0.01 µg/ml for 10 min). Intensities of anti-puromycin antibody are indicative for overall protein synthesis. Data were normalized to total protein (Ponceau S). Control cells pre-treated with protein synthesis inhibitor cycloheximide (CHX; 70 µM for 15 min) are shown as a control (n = 9 - 10; bars indicate mean ± SEM; *$p<0.05$, **$p<0.01$, ***$p<0.001$, ****$p<0.0001$ in a one-way ANOVA followed by *post hoc* Šídák's test; scale bars are 10 µm; FI = fluorescence intensity). **n, o** Western blots of RIPA soluble (S) and insoluble (I) TDP-43 of untreated (**n**) and sodium arsenite treated (50 µM for 30 min; **o**) HAP1 FXP-ko cells. GAPDH is shown as a marker of the soluble fraction. The horizontal line in (**n**) indicates that data from FXR2-ko cells is from a different blot.

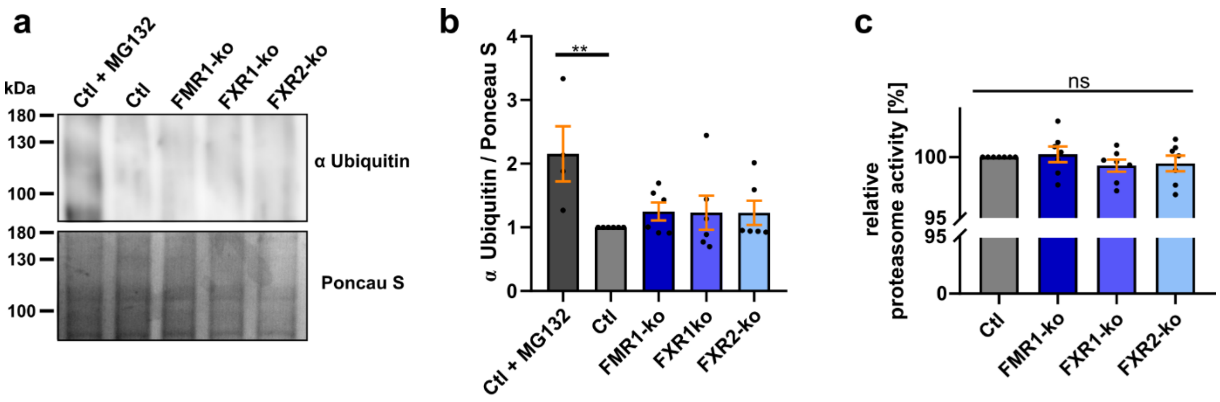

Supplementary Fig. S6 FXP loss has no effect on global protein ubiquitylation and proteasome activity. **a, b** Representative image (**a**) and quantification (**b**) of anti-ubiquitin Western blots of HAP1 cells. Control cells treated with proteasome inhibitor MG132 (20 µM for 4 h) are shown as a control. Data were normalized to total protein (Ponceau S; n = 4 - 6). **c** Proteasome activity measured by degradation of a fluorescent substrate in HAP1 cell lysates using 1 mg of total protein per sample (n = 7, measured in triplicates each; bars indicate mean ± SEM; ***p*<0.01 in a one-way ANOVA followed by *post hoc* Šídák's test).

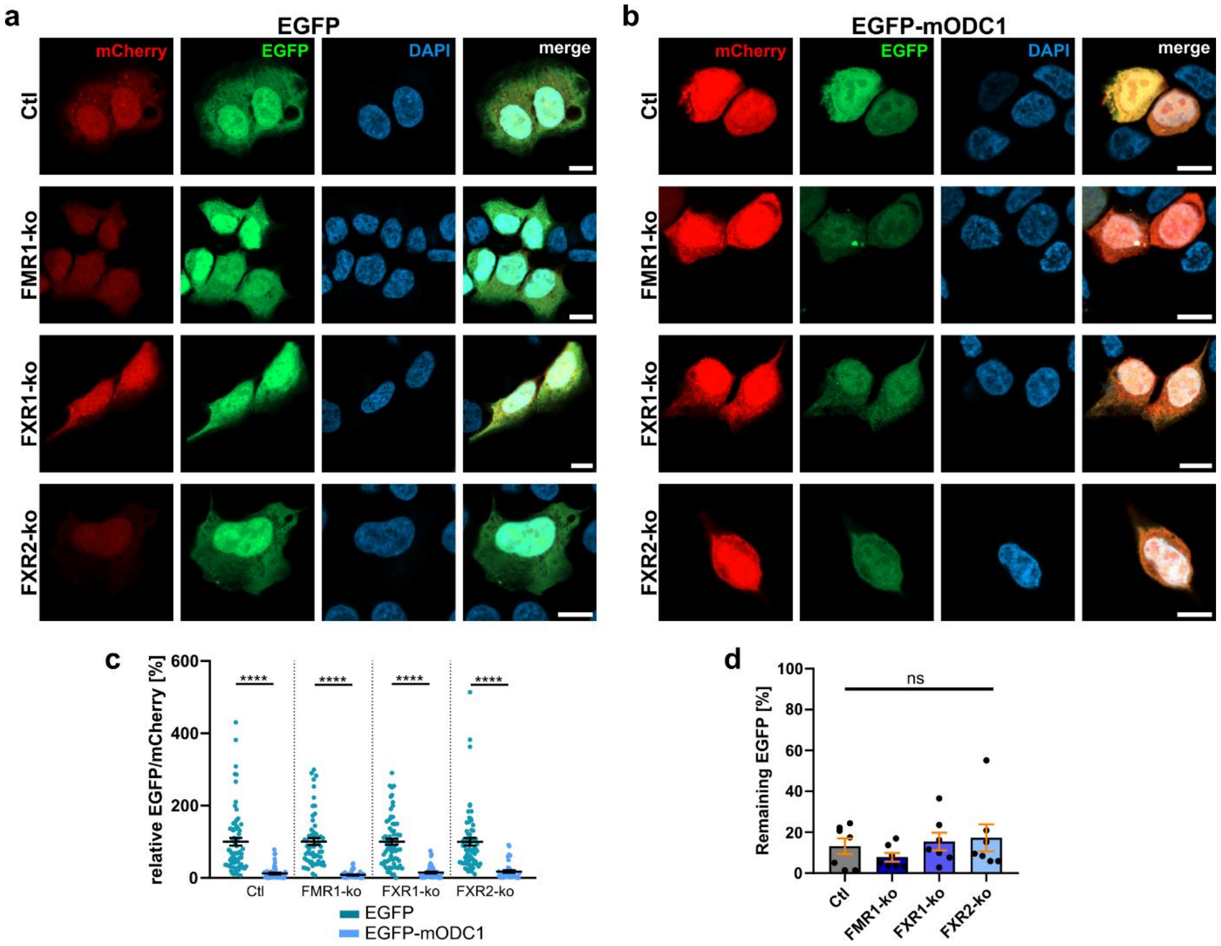

Supplementary Fig. S7 FXP loss has no effect on ubiquitin-independent proteasomal protein degradation in HAP1 cells. **a, b** Representative images of HAP1 cells transfected with plasmids coding for EGFP (**a**) or EGFP fused to the 44 C-terminal amino acids of mouse Ornithine decarboxylase 1 (EGFP-mOdc1; **b**) 48 h post transfection. **c** Normalized fluorescence intensities of EGFP and EGFP-mOdc1 in the respective HAP1 cell lines. mCherry encoded in the same plasmids was used to normalize for transfection efficiencies (n = 50 images from 7 independent experiments). **d** Comparison of fluorescence intensities of EGFP-mOdc1 for each independent experiment relative to EGFP from the data shown in **c** (bars indicate mean ± SEM; ****p<0.0001 in an unpaired, two-tailed Student's t-test (**a**); ns refers to a non-significant one-way ANOVA in **d**; scale bars are 10 µm).

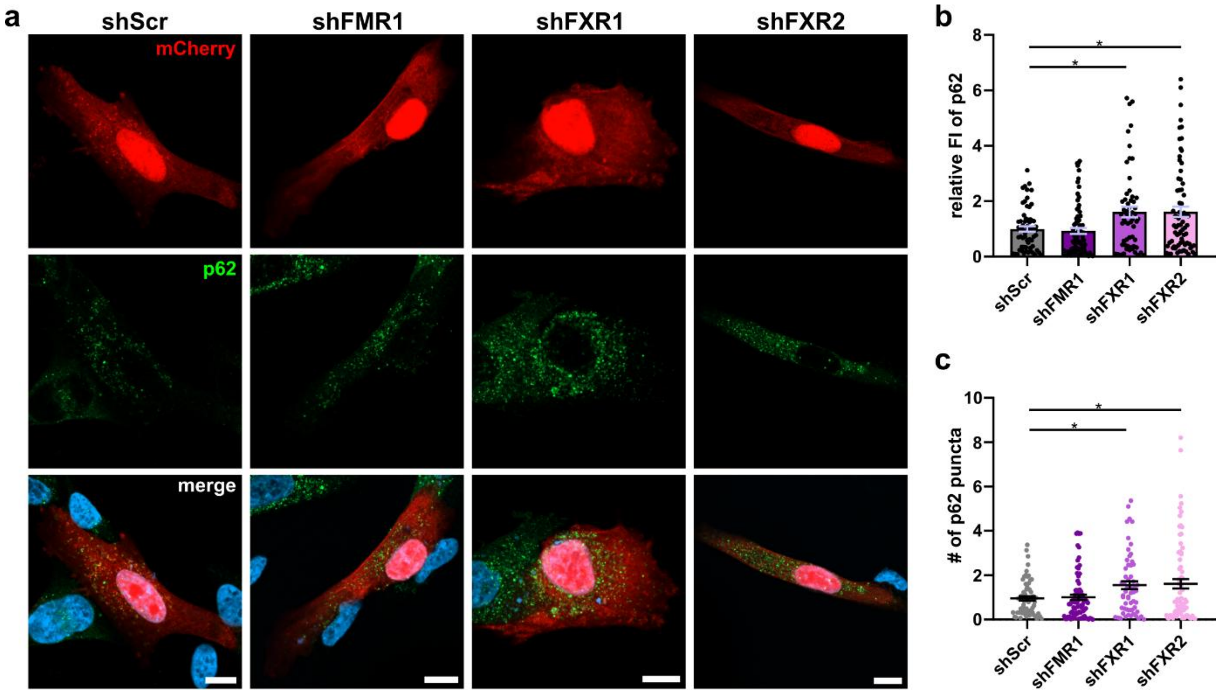

Supplementary Fig. S8 p62 accumulation indicates defects in autophagy upon knockdown of FXR1 or FXR2 in SH-SY5Y cells. **a-c** Representative images (**a**) and quantification of p62 fluorescence intensity (**b**) and puncta (**c**) of SH-SY5Y cells transfected with indicated shRNAs for 72 h. mCherry fluorescence was used to normalize for transfection efficiencies (n = 60 - 75 cells from 3 independent experiments; bars indicate mean ± SEM; *p<0.05 in a one-way ANOVA followed by *post hoc* Šídák's test; scale bars are 10 µm; FI = fluorescence intensity).

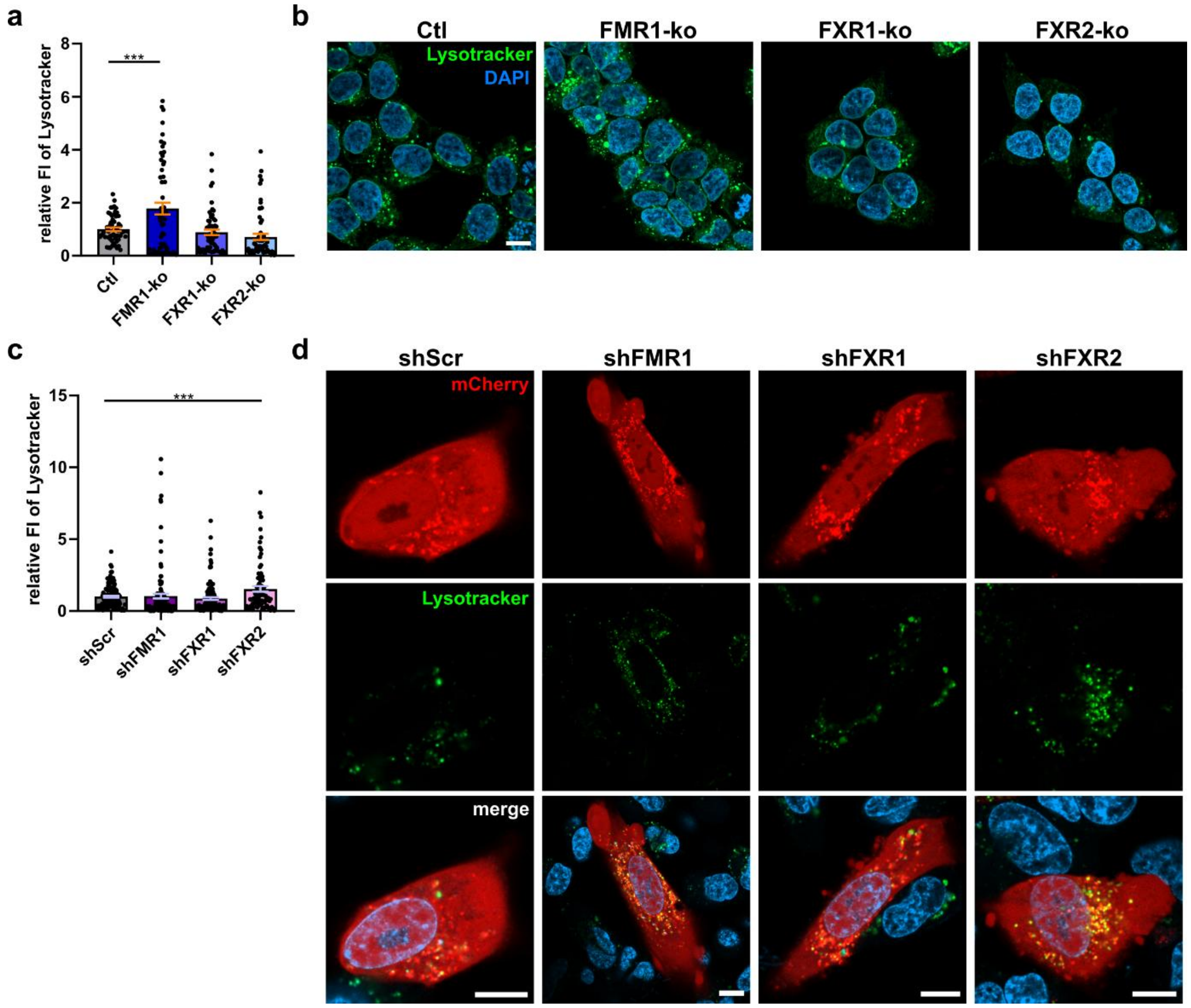

Supplementary Fig. S9 Knockout of FMR1 (HAP1) or knockdown of FXR2 (SH-SY5Y) increases Lysotracker signals. **a, b** Quantification of Lysotracker fluorescence intensity (**a**) and representative live cell images (**b**) of HAP1 cell lines (n = 58 - 68 images from 5 independent experiments). **c, d** Quantification of Lysotracker fluorescence intensity (**c**) and representative images (**d**) of FXP-kd SH-SY5Y cells in live cell imaging 72 h post transfection of respective shRNAs. mCherry fluorescence was used to normalize transfection efficiencies (n = 80 - 130 cells from 4 independent experiments; bars indicate mean ± SEM; ***p<0.001 in a one-way ANOVA followed by *post hoc* Šídák's test; scale bars are 10 µm; FI = fluorescence intensity).

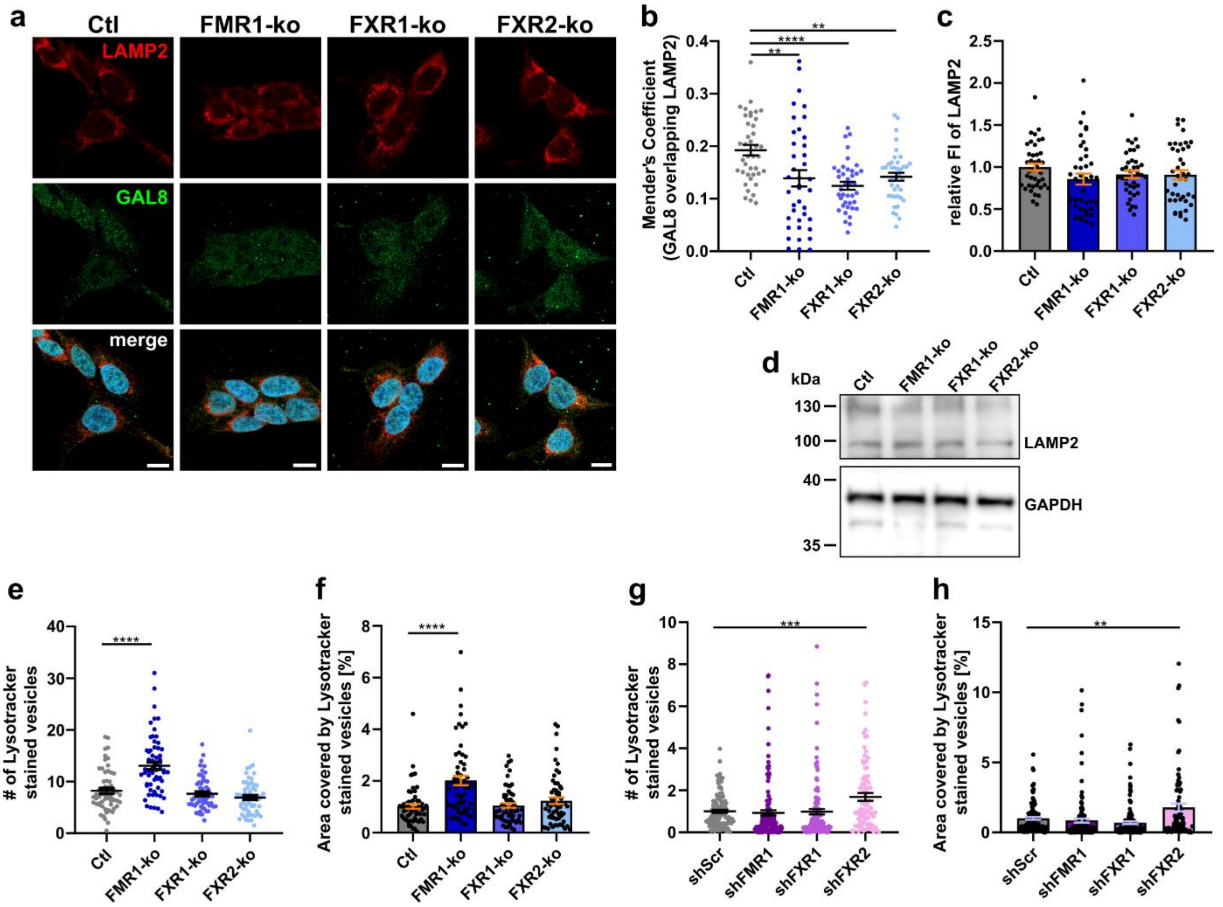

Supplementary Fig. S10 Analyses of lysosomal damage in HAP1 cells and re-analyses of lysotracker signals**. a, b** Representative images (**a**) and quantification (**b**) of co-localization of lysosomal marker LAMP2 and GAL8 (n = 70 images from 7 independent experiments). **c** Quantification of fluorescence intensities of LAMP2 (n = 40 images from 4 independent experiments). **d** Western blot of LAMP2 in HAP1 cells. **e, f** Re-analyses of Lysotracker signals in HAP1 cells (data from Supplementary Fig. S9). Numbers of vesicles (**e**) and areas covered by lysosomes (**f**) were determined. **g, h** Re-analyses of Lysotracker signals in SH-SY5Y cells (data from Supplementary Fig. S9). Numbers of vesicles (**g**) and areas covered by lysosomes (**h**) were determined (bars indicate mean ± SEM; ***p*<0.01; ****p*<0.001; *****p*<0.0001 in a one-way ANOVA followed by *post hoc* Šídák's test; scale bars are 10 µm; FI = fluorescence intensity).

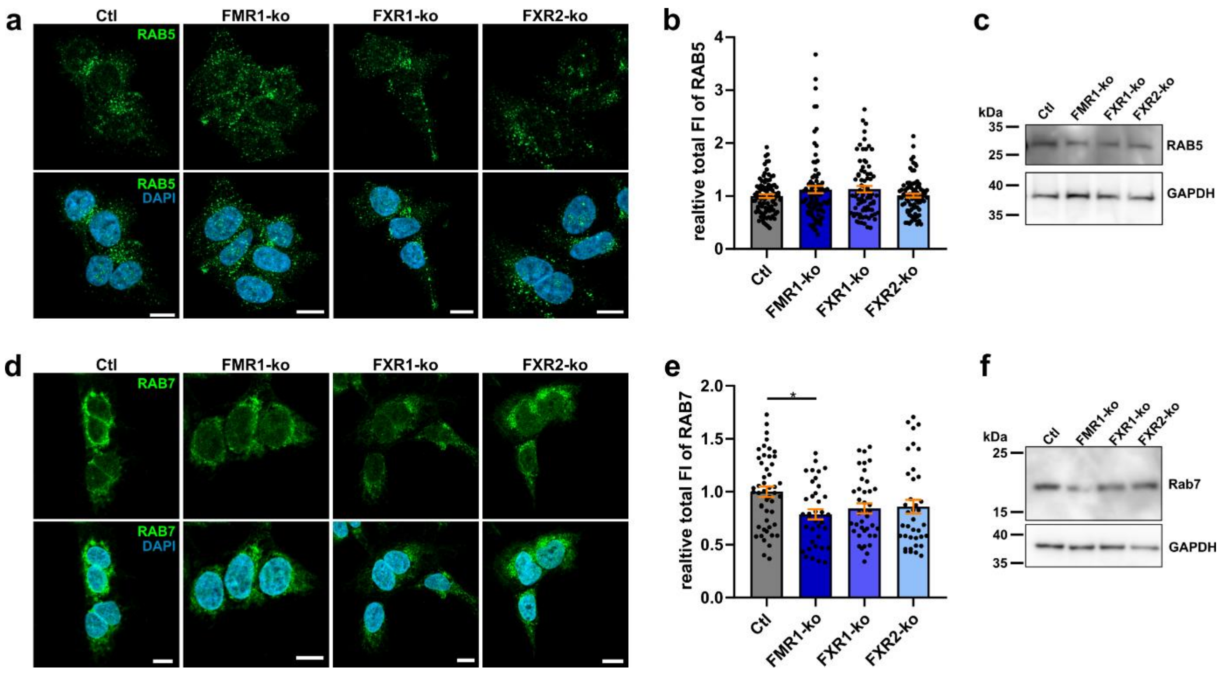

Supplementary Fig. S11 Analyses of early and late endosomes in HAP1 FXP-ko cells. **a-c** Representative images (**a**) and quantification of fluorescence intensity (**b**) of early endosomal marker RAB5. A Western blot of RAB5 is shown in (**c**). **d-f** Representative images (**d**) and quantification of fluorescence intensity (**e**) of late endosomal marker RAB7. A Western blot of RAB7 is shown in (**f**). (RAB5: n = 67 - 91 images from 6 – 7 independent experiments; RAB7: n = 37 - 39 images of 3 independent experiments; bars indicate mean ± SEM; *p<0.05 in a one-way ANOVA followed by *post hoc* Šídák's test; scale bars are 10 µm; FI = fluorescence intensity).

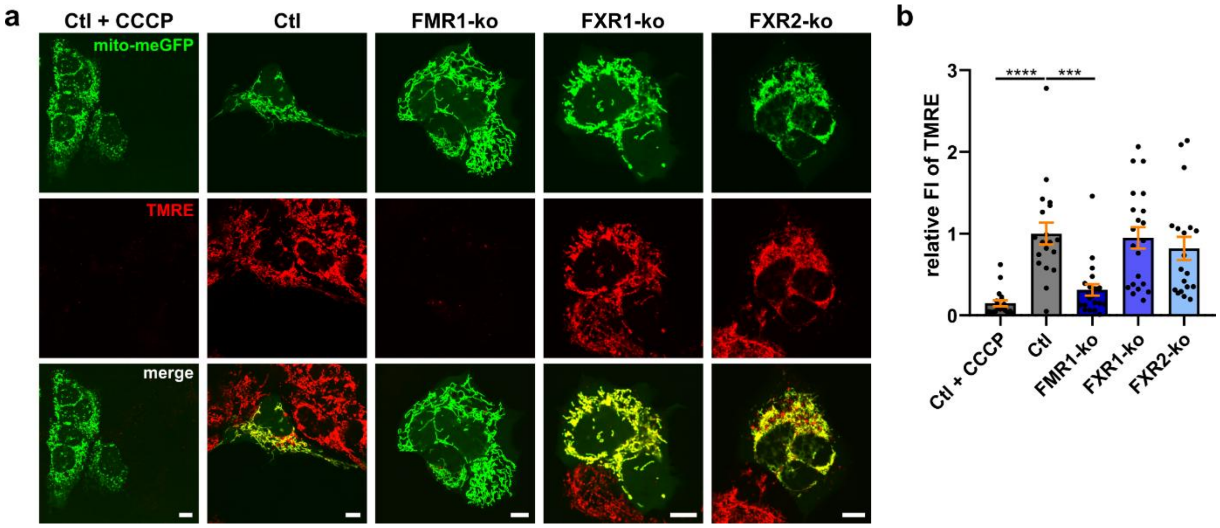

Supplementary Fig. S12 Loss of FMR1 severely decreases mitochondrial membrane potential in HAP1 cells. **a, b** Representative live cell images of HAP1 cells stained with TMRE (**a**) and respective quantification of fluorescence intensity of TMRE (**b**). Cells were transfected with mito-meGFP 30 h before analyses for staining of mitochondria to facilitate selection of regions-of-interest for measuring TMRE intensities. Control cells treated with CCCP (100 µM for 2 h) are shown as a control (n = 19 - 21 images from 3 independent experiments; bars indicate mean ± SEM; ***p<0.001; ****p<0.0001 in a one-way ANOVA followed by *post hoc* Šídák's test; scale bars are 10 µm; FI = fluorescence intensity).

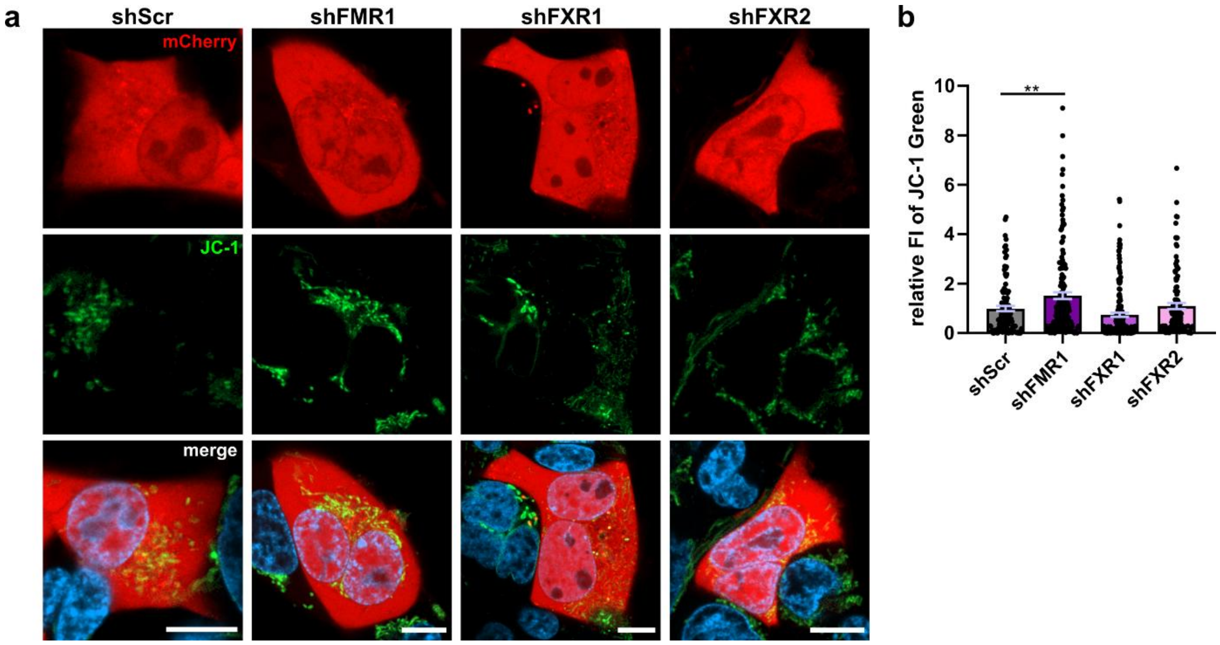

Supplementary Fig. S13 Knockdown of FMR1 decreases mitochondrial membrane potential in SH-SY5Y cells. **a, b** Representative live cell images (**a**) and quantification of fluorescence intensities of JC-1 dye (**b**; green channel only) 72 h post transfection of respective shRNAs (n = 110 - 176 cells from 3 independent experiments; bars indicate mean ± SEM; $^{**}p<0.01$ in a one-way ANOVA followed by *post hoc* Šídák's test; scale bars are 10 µm; FI = fluorescence intensity).

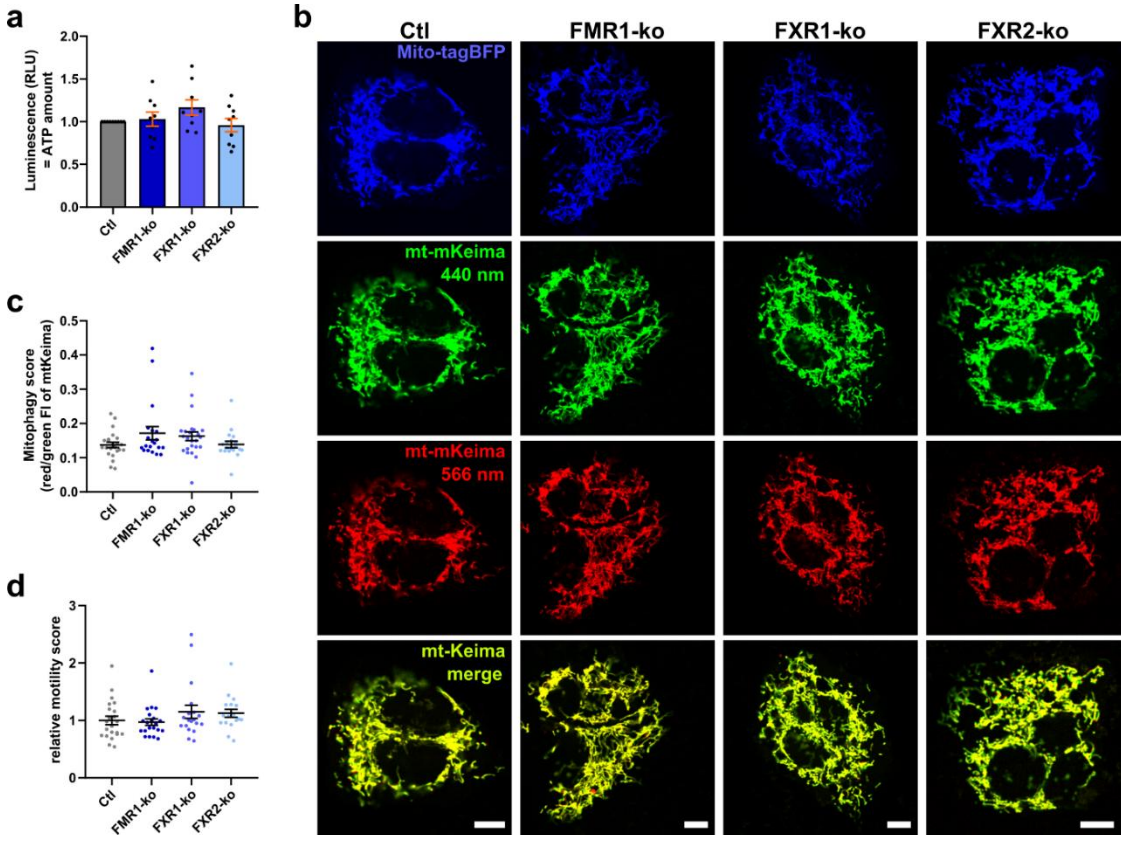

Supplementary Fig. S14 FXP loss has no effect on ATP level, mitochondrial motility or mitophagy in HAP1 cells. **a** Luminescence of Ultra-Glo™ rLuciferase (CellTiter-Glo® 2.0 Assay Kit; Promega) indicative for cellular ATP level. Same numbers (1 x 10^4) of cells from each cell line were used directly after cell counting (n = 9, measured in triplicates each; non-significant in a one-way ANOVA). **b** Representative live cell images of HAP1 cells 30 h post transfection of mt-mKeima. Mt-mKeima excited at 440 nm (mitochondrial) and at 566 nm (lysosomal) are shown. Mito-tagBFP was co-transfected along mt-mKeima to facilitate definition of regions-of-interest (scale bars are 10 µm). **c** Quantification of mt-mKeima fluorescence excited at 440 nm and 566 nm. The ratio of lysosomal (566 nm) to mitochondrial (440 nm) fluorescence is shown (n = 19 - 24 images from 3 independent experiments; bars indicate mean ± SEM; non-significant in a one-way ANOVA; FI = fluorescence intensity). **d** Mitochondrial motility scores assessed in HAP1 cell lines co-transfected with EGFP (visualization of cells) and mito-Raspberry-7 (visualization of mitochondria) 30 h post transfection. Live cell videos were quantified using the QuoVadoPro Fiji Plugin (see material and methods; n = 18 - 22 videos from 4 independent experiments; non-significant in a one-way ANOVA).

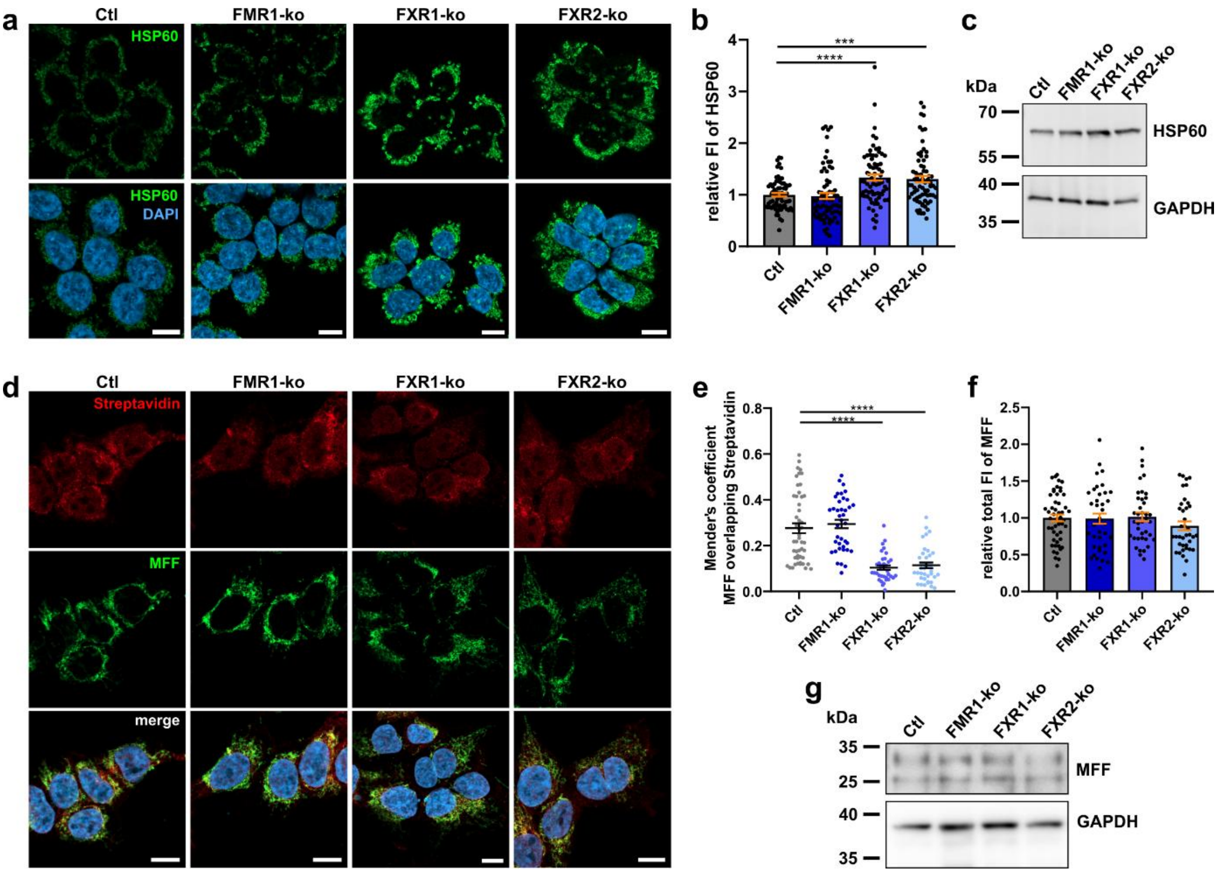

Supplementary Fig. S15 FXR1 and FXR2 may be involved in mitochondrial fission in HAP1 cells. **a, b** Representative images (**a**) and quantification of fluorescence intensities of HSP60 (**b**; n = 71 - 73 images from 7 independent experiments). **c** Western blot of HSP60 in HAP1 cells. **d** Representative images of HAP1 cells stained for mitochondria (Streptavidin) and mitochondrial fission factor (MFF). **e** Co-localization analyses of MFF and Streptavidin. **f, g** Relative fluorescence intensity (**f**) and Western blot (**g**) of MFF (n = 38 - 39 images from 3 independent experiments; bars indicate mean ± SEM; ***p<0.001; ****p<0.0001 in a one-way ANOVA followed by *post hoc* Šídák's test; scale bars are 10 μm; FI = fluorescence intensity).

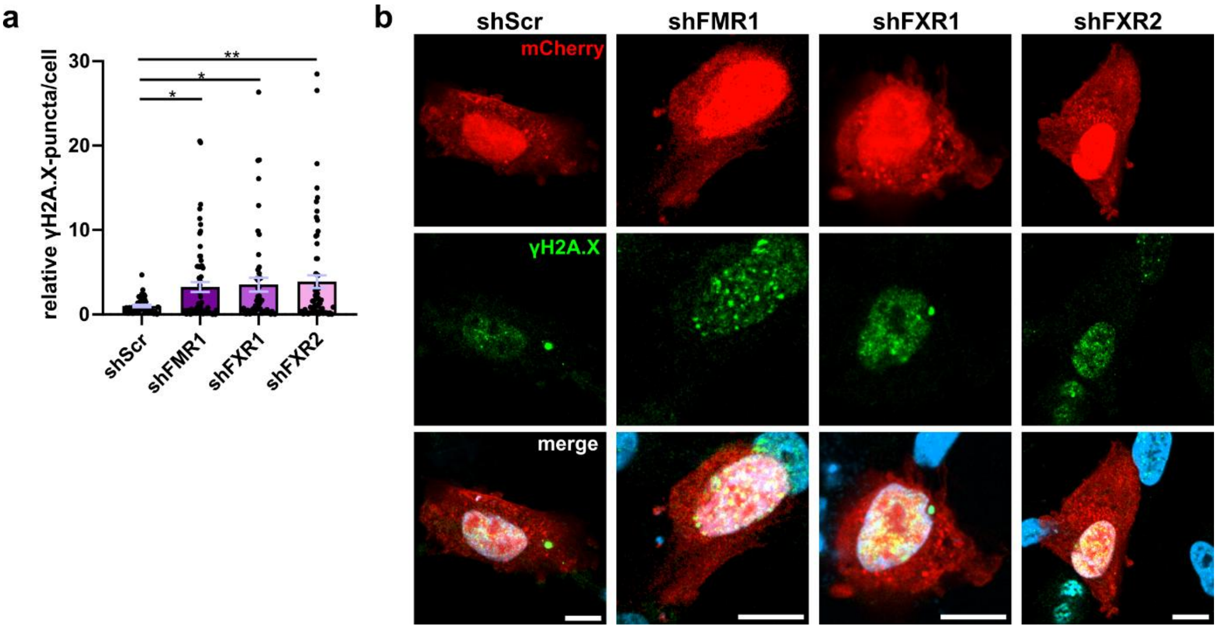

Supplementary Fig. S16 Knockdown of FXPs induces DNA damage in SH-SY5Y cells. **a, b** Quantification (**a**) of γH2A.X puncta per nucleus in SH-SY5Y cells 72 hours post-transfection of respective shRNAs, and respective representative images (**b**; n = 50 - 67 cells from 3 independent experiments; bars indicate mean ± SEM; *p<0.05; **p<0.01 in a one-way ANOVA followed by *post hoc* Šídák’s test; scale bars are 10 μm).

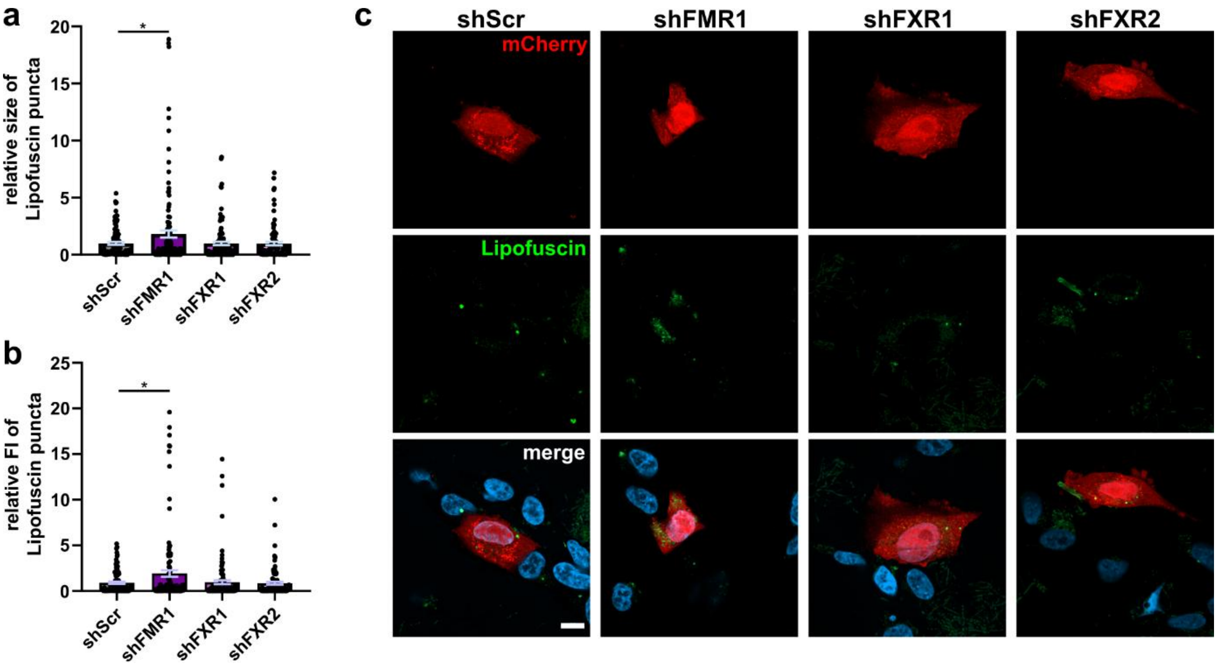

Supplementary Fig. S17 Knockdown of FMR1 induces lipofuscin accumulation in SH-SY5Y cells. **a-c** Quantification of relative size (**a**) and fluorescence intensity (**b**) of autofluorescent lipofuscin puncta, and respective representative images (**c**) of SH-SY5Y cells 72 h post transfection of respective shRNAs (n = 96 - 130 cells from 8 independent experiments; bars indicate mean ± SEM; *p<0.05 in a one-way ANOVA followed by *post hoc* Šídák’s test; scale bar is 10 µm).

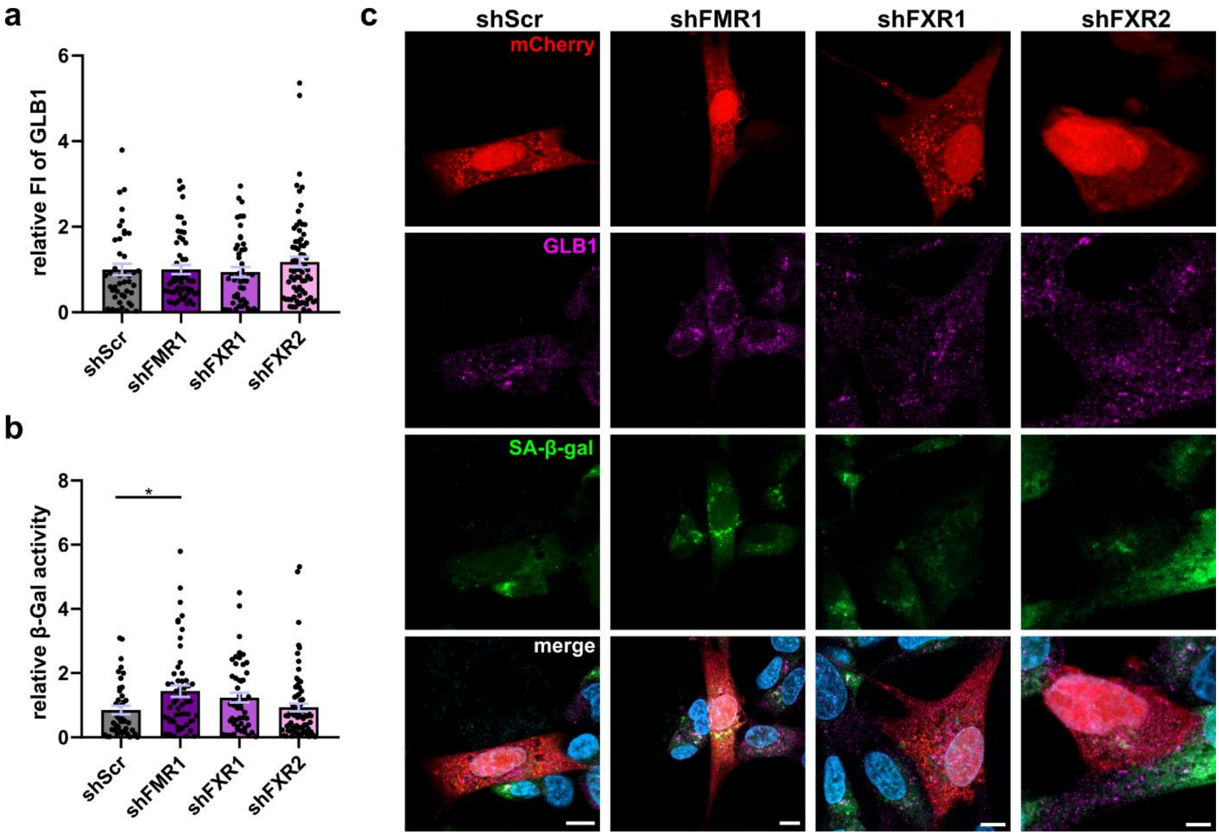

Supplementary Fig. S18 Knockdown of FMR1 leads to increased ß-Galactosidase activity in SH-SY5Y cells. **a, b** Quantification of ß-Galactosidase (GLB1) fluorescence intensities (**a**) and senescence-associated ß-Galactosidase activity (SA-ß-gal; **b**) in SH-SY5Y cells 72 h post transfection of respective shRNAs. **c** Respective representative images of stained SH-SY5Y cells (n = 43 - 72 cells from 3 independent experiments; bars indicate mean ± SEM; *$p<0.05$ in a one-way ANOVA followed by *post hoc* Šídák's test; scale bar is 10 µm; FI = fluorescence intensity).

Supplementary Tables

Supplementary Table S1: HAP1 cell lines (Horizon Discovery) used in this study.

Cell line	Description	Catalogue #
control	Human parental HAP1 control cell line	C631
FMR1 knockout	23 bp deletion in exon 5 of *FMR1*	HZGHC005871c008
FXR1 knockout	8 bp deletion in exon 8 of *FXR1*	HZGHC005627c009
FXR2 knockout	32 bp deletion in exon 5 of *FXR2*	HZGHC007894c012

Supplementary Table S2: Forward Sequences of shRNAs.

shRNA	Sequence
shScrmabled (shScr)	*CCGGGGCGCGATAGCGCTAATAATTCTCGAGAATTATTAGCGCTATCGCGCCTTTTTG*
shFMR1	*CCGGCGAGATTTCATGAACAGTTTACTCGAGTAAACTGTTCATGAAATCTCGTTTTTG*
shFXR1	*CCGGGAAACGGAATCTGAGCGTAAACTCGAGTTTACGCTCAGATTCCGTTTCTTTTTG*
shFXR2	*CCGGGATGTGCCTTTATCCTCTAATCTCGAGATTAGAGGATAAAGGCACATCTTTTTG*

Supplementary Table S3: Plasmids used in this study.

Plasmid	Description	Reference
shRNAs	pcDNA3 (Invitrogen) backbone; CMV promotor replaced by human U6 promoter, followed by the respective shRNA-Sequence; Neomycin resistance was replaced with mCherry for identification of transfected cells.	This study
pCH3_EGFP<br>pCH3_EGFP-mODC	pcDNA3 backbone (Invitrogen) coding for EGFP or EGFP-mOdc1 (amino acid 418-461); Neomycin resistance was replaced with mCherry to provide a transfection control.	This study
pCI-neo EGFP<br>pCI-neo Fluc EGFP<br>pCI-neo FlucSM EGFP<br>pCI-neo FlucDM EGFP	Plasmids coding for EGFP alone, or EGFP fused to american firefly luciferase wild type, single mutant (SM; R188Q), or double mutant (DM; R188Q, R261Q), respectively	[1]
NanoLuc (Y18X)<br>NanoLuc (R162S)<br>Firefly luciferase	NanoLuc plasmids were generated from pNL1.1.PGK [Nluc/PGK] (Promega). The plasmid coding for firefly luciferase is pGL3/SV40 (Promega).	[2]
mito-Raspberry-7	Mitochondrial marker to track mitochondrial movement	Unpublished (Addgene 55931)
pEGFP-N1	Plasmid coding for EGFP used to visualize cells.	[3]
pHAGE mt-mKeima	Mitophagy sensor	[4]
mito-tagBFP	Mitochondrial marker	[5]
mito-meGFP	Mitochondrial marker	Unpublished (Addgene 172481)

Supplementary Table S4: Antibodies used for Western Blotting and ICC.

Name	Manufacturer	Catalogue #	dilution used in	
			WB	ICC
**Primary antibody**				
anti-FMR1	Atlas Antibodies	HPA050118	1:1000	1:300
anti-FXR1	Atlas Antibodies	HPA018246	1:1000	1:300
anti-FXR2	Thermo Fisher Scientific	MA1-16767	1:1000	1:300
anti-GAPDH	Proteintech	10494-I-AP	1:20000	
anti-rRNA (Y10B)	Invitrogen	MA1-16628		1:300
anti-Fibrillarin	Sigma-Aldrich	MABE1154	1:500	1:250
anti-CDKN2A/ p16INK4a	Invitrogen	PA5-20379	1:1000	
anti-CDKN1A/p21	abcam	ab227443	1:3000	
anti-TP53	Proteintech	10442-1-AP	1:2000	
anti-phospho-TP53	Cell Signaling Technology	9284	1:1000	
anti-Puromycin	merck	ZMS1016-25UL	1:4000	
anti-LAMP2	Proteintech	66301-1-Ig	1:1000	1:500
anti-Gal-8	Thermo Fisher Scientific	MA5-34693		1:500
anti-RAB5	Cell Signaling Technology	3547S	1:1000	1:200
anti-RAB7	abcam	ab50533	1:1000	1:500
anti-p62 (SQSTM1)	MBL life science	PM045	1:1000	1:500
anti-LC3B	Cell Signaling Technology	2275	1:1000	
anti-Ubiquitin	Thermo Fisher Scientific	89899	1:500/1000	
anti-TOM20	Sigma-Aldrich	MABT166	1:1000	1:500
anti-HSP60	Stress Marq	SPC-105D	1:1000	1:100
anti-MFF	Proteintech	17090-1-AP	1:5000	1:500
anti-γH2A.X	Sigma-Aldrich	05-636-25UG		1:500
anti-GLB1	Thermo Fisher Scientific	PA5-65272	1:500	1:100
anti-TDP-43	Proteintech	12892-1-AP	1:1000	
**Secondary antibody**				
anti-mouse HRP	Thermo Fisher Scientific	G-21040	1:5000	
anti-rabbit HRP	Thermo Fisher Scientific	G-21234	1:5000	
anti-rabbit A488	Thermo Fisher Scientific	A-11008		1:500
anti-mouse A546	Thermo Fisher Scientific	A-11003		1:500
anti-mouse A647	Thermo Fisher Scientific	A-31571		1:500
anti-rabbit A647	Thermo Fisher Scientific	A-31573		1:500

Supplementary Table S5: Kits and dyes used in this study.
Modifications of the protocol provided by the manufacturer are indicated.

Kit/Dye	Final concentration	Manufacturer	Catalogue #
Autophagy Assay Kit	-	abcam	139484
CellEvent Senescence Green Detection Kit	-	Invitrogen	C10850
CellTiter-Glo® 2.0 Assay *	-	Promega	G9241
Proteasome Activity Assay Kit	-	abcam	ab107921
PROTEOSTAT® Aggresome Detection Kit	-	Enzo Life Sciences	ENZ-51035
Streptavidin A 647 conjugate	2 µg/ml	Thermo Fisher Scientific	S21374
TPE-MI	50 µM	Sigma-Aldrich	797316-25MG
TMREred	0.05 µM	Sigma-Aldrich	87917

* This kit was used to measure ATP level in HAP1 cells. Here, for each independent experiment, luminescence of $1*10^4$ suspended cells (directly after cell counting) was measured in triplicates on a FLUOstar Omega Plate reader (BMG Labtech).

Supplementary Table S6: QuantiTect Primer Assays (Qiagen; #249900).

Primer Assay	GeneGlobe ID
Hs_TBP_1_SG	QT00000721
Hs_TP53_1_SG	QT00060235
Hs_CDKN1A_1_SG	QT00062090
Hs_CDKN2A_1_SG	QT00089964
Hs_CDKN2A_va.1_SG	QT00998452
Hs_RRN18S_1_SG	QT00199367

Supplementary Table S7: Excel file total proteome

Supplementary Table S8: Excel file insoluble proteome

Supplementary References

1. Gupta R, Kasturi P, Bracher A, et al (2011) Firefly luciferase mutants as sensors of proteome stress. Nature Methods 2011 8:10 8:879–884. https://doi.org/10.1038/nmeth.1697

2. Hartmann M, Neher L, Grupp B, et al (2025) Development of a highly sensitive method to detect translational infidelity. Biol Methods Protoc 10:. https://doi.org/10.1093/BIOMETHODS/BPAF008

3. Pekkurnaz G, Trinidad JC, Wang X, et al (2014) Glucose regulates mitochondrial motility via Milton modification by O-GlcNAc transferase. Cell 158:54–68. https://doi.org/10.1016/J.CELL.2014.06.007

4. Vargas JNS, Wang C, Bunker E, et al (2019) Spatiotemporal Control of ULK1 Activation by NDP52 and TBK1 during Selective Autophagy. Mol Cell 74:347-362.e6. https://doi.org/10.1016/J.MOLCEL.2019.02.010

5. Friedman JR, Lackner LL, West M, et al (2011) ER tubules mark sites of mitochondrial division. Science 334:358–362. https://doi.org/10.1126/SCIENCE.1207385
